# Evaluation of neuroprotective and anti-amnesic effects of *Elaeagnus umbellata* Thunb. On scopolamine-induced memory impairment in mice

**DOI:** 10.1186/s12906-020-02942-3

**Published:** 2020-05-12

**Authors:** Nausheen Nazir, Muhammad Zahoor, Mohammad Nisar, Nasiara Karim, Abdul Latif, Sajjad Ahmad, Zia Uddin

**Affiliations:** 1 Department of Biochemistry, University of Malakand, Chakdara Dir (L), Khyber Pakhtunkhwa, Pakistan; 2 Department of Botany, University of Malakand, Chakdara Dir (L), Khyber Pakhtunkhwa, Pakistan; 3 Department of Pharmacy, University of Malakand, Chakdara Dir (L), Khyber Pakhtunkhwa, Pakistan; 4 Department of Chemistry, University of Malakand, Chakdara Dir (L), Khyber Pakhtunkhwa, Pakistan; 5 Department of Pharmacy, Sarhad University of Information Technology, Peshawar, Pakistan; 6 Department of Pharmacy, COMSATS University Islamabad, Abbottabad Campus, Abbottabad, Pakistan

**Keywords:** *Elaeagnus umbellata*, Amnesia, Cholinesterases, Scopolamine, Y-maze test, Novel object recognition test, Molecular docking

## Abstract

**Background:**

*Elaeagnus umbellata* is abundantly found in Himalayan regions of Pakistan which is traditionally used to treat various health disorders. However, the experimental evidence supporting the anti-amnesic effect is limited. Therefore the study was aimed to evaluate the prospective beneficial effect of *E. umbellata* on learning and memory in mice.

**Objectives:**

To assess neuroprotective and anti-amnesic effects of *E. umbellata* fruit extracts and isolated compounds on the central nervous system.

**Methods:**

Major phytochemical groups present in methanolic extract of *E. umbellata* were qualitatively determined. The total phenolic and flavonoid contents were also determined in extract/fractions of *E. umbellata*. On the basis of in vitro promising anticholinesterases (AChE & BChE) and antioxidant activities observed for CHF. Ext and isolated compound-**I** (Chlorogenic acid = CGA), they were further evaluated for learning and memory in normal and scopolamine-induced cognitive impairment in mice using memory behavioral tests such as the Y maze and Novel object recognition using standard procedures. The test sample were further assessed for in vivo anticholinesterases (AChE & BChE) and DPPH free radical scavenging activities in mice brain sample and finally validated by molecular docking study using GOLD software.

**Results:**

The extract/fractions and isolated compounds were tested for their anticholinesterase and antioxidant potentials. The CHF. Ext and CGA showed maximum % inhibition of tested cholinesterases and free radicals. The CHF. Ext and CGA reversed the effects of scopolamine in mice. The CHF. Ext and CGA significantly increased the alternate arm returns and % spontaneous alteration performance while escape latency times (second) significantly decreased in Y maze test. The CHF. Ext and CGA significantly increased the time spent with novel object and also increased the discrimination index in the Novel object recognition test. Furthermore, molecular docking was used to validate the mechanism of cholinesterases inhibition of isolated compounds.

**Conclusion:**

The data obtained from behavioral and biochemical studies (AChE/BChE and DPPH/ABTS inhibition) have shown that *E. umbellata* possessed significant memory enhancing potency. These results suggest that *E. umbellata* extract possess potential antiamnesic effects and amongst the isolated compounds, compound **I** could be more effective anti-amnesic therapeutics. However, further studies are needed to identify the exact mechanism of action.

**Graphical abstract:**

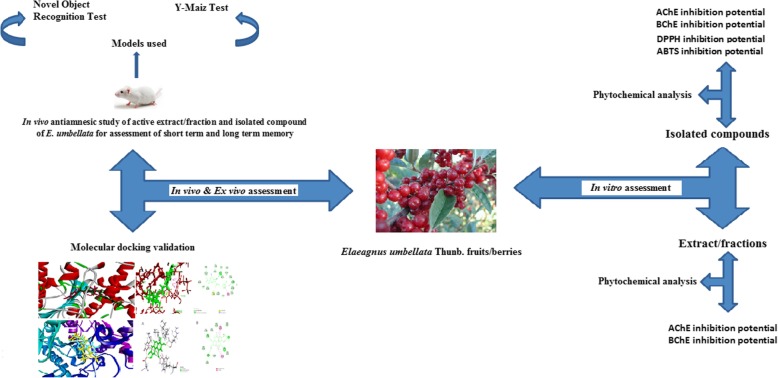

## Background

Alzheimer’s disease (AD) is a neurodegenerative fatal brain disorder that causes both physical and mental deterioration in human leading to death of an individual suffering from this disease. According to World Alzheimer’s organization report 2015, globally the number of individuals affected by AD and other forms of dementia are about 46.85 million. They have also predicted that this number will be doubled by 2030 which would further increase to three folds of the existing level by 2050 [1, 2]. Clinically, Alzheimer’s disease can be characterized by progressive memory deficits, inappropriate behaviour, and cognitive dysfunctions such as learning impairment and amnesia being the most remarkable age-related alterations [3]. Pathologically, AD has been characterized by loss of the cholinergic neurons, extracellular deposition of β-amyloid peptide and low level of neurotransmitter acetyl choline (ACh) [4–6]. Acetyl cholinesterase (AChE) and butyryl cholinesterase (BChE) enzymes cause degradation of ACh which produces cholinergic deficits. Inhibitions of these two enzymes are considered as a best strategy to treat AD, dementia, and Parkinson^’^s disease [7]. However, the ultimate cause of AD is still a mystery, there are several hypothesis about its causes [8]. Amnesia is a condition of severe disruption of memory from the impairment of the hypothalamus, thalamus and the surrounding cortical structures without deficits in cognition, attention, awareness or judgment [9–11]. Scientists all over the world are exploring novel compounds which could attenuate the neurological disorders and amnesia that may probably be due to the vulnerability of the brain cells to increased production of free radicals [12, 13].

Antioxidant remedy has proved to be successful in the amelioration of cognitive dysfunction and behavioral insufficiency in patients with mild to moderate AD. Natural products are the major source of antioxidants which delay the development of AD and attenuate neuronal cell death [14, 15].

Medicinal plants received much attention due to the presence of important bioactive secondary metabolites, such as alkaloids, terpenoids, carotenoids, poly phenols, and flavonoids [16, 17]. Among the medicinal plants *E. umbellata* Thumb. a wild spiny branched shrub that belongs to *Elaeagnaceae* family, is a very important plant as they have berries fruit [18]. Apart from their edibility, different parts of the *E. umbellata* have been used in folk medicine as anti-inflammatory, muscle relaxant, antipyretic, analgesic, astringent, antiulcer, antidiabetic, anti-diarrheal, as a tonic to cure coughs and pulmonary complications [19–23]. Several studies have shown that regular consumption of polyphenolic rich barriers fruits are associated with delayed Alzheimer’s disease and other brain related disorders because of their anti-oxidant, anti-inflammatory, and anti-proliferative properties [24–26].

In this investigational study, an attempt has been made to establish a link between the neuroprotective beneficial effects of berries with its phytoconstituents. Chlorogenic acid and ellagic acid are the polyphenolic compounds that have shown strong neuroprotective and cognitive enhancement effects on scopolamine-induced amnesia and anti-oxidative activities in animal models [27–29]. Keeping in mind, the mentioned properties of isolated compound, chlorogenic acid it was expected that it could be a leading therapeutic having impact on memory disorders and would have a positive impact on human health which would be an effective remedy for the treatment of degenerative diseases.

## Methods

### Chemicals

All the chemical used were of analytical grade with the exception of HPLC solvents that were of HPLC grade. The DTNB (5, 5-dithio-bis-nitrobenzoic acid), Quercetin, Streptozotocin, PNPG, Galantamine (*Lycoris Sp.*), Acetyl cholinesterase (*Electron-eel* Type, VI-S), butyryl cholinesterase (lot No SLBPO912V), 3,5- Dinitrosalicylic acid (lot No D2401QEI), DPPH, ABTS, Ascorbic acid, Acetylthiocholine-iodide, Butyrylthiocholine-iodide, Gallic acid & Folin-Ciocalteu regent were obtained from Sigma-Aldrich, Germany.

### Plant material collection

The fruits of *E. umbellata* Thunb. were collected from the hilly areas of Kalam, Malakand Division, Khyber Pakhtunkhwa, Pakistan, in August–September 2016. The plant sample was identified by plant taxonomist; Prof. Mehboob-ur-Rahman, PGC. Swat, Khyber Pakhtunkhwa, Pakistan. The plant specimens were deposited in the Botanical Garden Herbarium, University of Malakand, Pakistan with voucher number BGH.UOM.154. The plant variety selected was a wild one therefore, permission was taken from Divisional Forest Officer, Kalam and Local administration.

### Extraction, fractionation and isolation of pure compounds

*E. umbellata* Thunb. fruits extract preparation and fractionation were carried out according to the method described in a previously published research article with slight modification [19]. About 10 kg of dried fruits were crushed through mechanical grinder into fine powders which were then macerated in 80% methanol for 14 days with periodical shaking. Filtration was carried out through muslin cloth followed by Whattman filter paper. The filtrates were converted into a semisolid mass under reduced pressure in the rotary evaporator (Schwabach: 4000; Heidolph-Laborota-Germany) at 40 °C. The semisolid mass was then solidified (750 g end yield) in open air. The solvent-solvent extraction method was used to get different fractions using different solvents starting from a low to high polarity. The final yield of *n*-hexane (Hex.Ext), chloroform (CHF.Ext), ethyl acetate (EtAc.Ext), *n*- butanol (But.Ext) and aqueous (Aq.Ext) fractions were 95, 210, 115, 90 and 220 g respectively. Being the most active fraction biologically (in-vitro analysis results), the CHF. Ext fraction was subjected further to isolation of bioactive compounds in pure state. The CHF. Ext fraction was mixed with silica gel slurry and then allowed to dry in air. The sample loaded silica was then carefully loaded to large silica gel column with internal diameter of 10 cm packed height 50 cm using a gradient of increasing polarity with total *n*-hexane to total ethyl acetate as mobile phase. The effluents of columns were separated into 15 fractions on the basis of TLC analysis for random isolation of compounds. Finally the narrow pen column was used to separate the active sub fraction and collect it into small vials. The effluents of pencil column were spotted on TLC plates with suitable solvent system. Fraction 11 (12.0 g) was re-separated by silica gel column chromatography in solvent system of *n*-hexane and ethyl acetate (3:7) to afford pure compound-**I** (11.2 mg). Fraction 9 (4.25 g) was re-separated by silica gel column chromatography in solvent system of *n*-hexane and ethyl acetate (2:8) to afford pure compounds**-II** (32.4 mg). Fraction 7 (8.0 g) give pure compounds**-III** (20 mg) in solvent system of *n*-hexane and ethyl acetate (4:6), while fraction 4 (11.22 mg) was re-separated by silica gel column chromatography in solvent system of *n*-hexane and ethyl acetate (6:4) to afford pure compounds**-IV** (4.20 mg). The vials with identical R_f_ values were mixed together and purified. After purification, the compound were characterized through different spectroscopic techniques like FTIR, HNMR, 13CNMR and Mass spectrometry. The purified compounds were screened for in-vitro anticholinesterase and in-vivo anti-amnesic activities.

### Preliminary screening of phytochemicals

Hydro methanolic extract (Met.Ext) of *E. umbellata* fruit was investigated to identify major phytoconstituents like flavonoids, alkaloids, glycosides, terpenoids, anthraquinone, tannins and pigments using reported assays [30].

### Estimation of total phenolic content

Previously reported methods [31, 32] were used to estimate the total phenolic contents (TPC) in extract/fractions like hydro methanolic extract, *n*-hexane, chloroform, ethyl acetate, *n-*butanol and aqueous fraction of *E. umbellata* fruit. The extract/fractions samples (100 μL), distilled water (500 μL), Folin-Ciocalteu reagent (100 μL) and 1000 μL Sodium Carbonate (7%) were mixed and allowed to stand for 90 min and finally at 760 nm absorbance was recorded through UV-Spectrophotometer against the blank solution containing the extraction solvent instead of the sample. Calibration curve was drawn for standard Gallic acid as well. From calibration curve, the TPC in samples were calculated and reported as mg GAE/g of dry sample averaged from 3 parallel measurements.

### Estimation of total flavonoid contents

According to previously reported methods [31, 32], the total flavonoid contents (TFC) was measured as mg QE/g of dry samples of extract/fractions like hydro methanolic extract, *n*-hexane, chloroform, ethyl acetate, *n-*butanol and aqueous fraction of *E. umbellata* fruit. A calibration curve was drawn for different dilution of standard quercetin. From each sample dilutions 100 μL were taken and mixed with 500 μL distilled water, 100 μL Sodium nitrate (5%), 150 μL Aluminium chloride (10%) and 200 μL Sodium hydroxide (1 M). After 5 min absorbance were recorded at 510 nm by UV-Spectrophotometer against a blank containing extraction solvent. All tests were achieved in triplicate.

### In-vitro anti-cholinesterase assay

Ellman assay [33] was used to evaluate *E. umbellata* fruit extracts and isolated compounds for their anticholinesterase potentials. About 205 μL extract/compound solutions and 5 μL of AChE (0.03 U/mL)/BChE (0.01 U/mL) along with catalyst DTNB (5 μL) were taken in a cuvette and kept at 30 °C in hot water bath for 15 min. After 15 min of incubation acetylthiocholine iodide or butyrylthiocholine iodide were added as a substrate (5 μL) to the mixture that resulted in yellow colouration (5-Thio-2-nitro benzoate anion colour). Then the absorbance was recorded at 412 nm through double beam spectrophotometer (Thermo electron-corporation; USA). As a negative control solution all the above mentioned components except extracts and isolated compounds were mixed in the mentioned order. Donepezil was used as a positive control. The same procedure mentioned above was used for reaction mixture of positive control and absorbance was measured at 412 nm. For each sample absorption was recorded for 4 min. Percent enzyme activity and inhibition potential of both enzymes were measured by the following formulas:

1 $$ V=\varDelta \kern0.5em Abs/\varDelta t $$

2 $$ \% enzyme\ activity\kern0.5em =\kern0.5em \frac{V}{V\max}\kern0.5em \times \kern0.5em 100 $$

3 $$ \% enzyme\ inhibition\kern0.5em =\kern0.5em 100\kern0.5em \hbox{-} \kern0.5em \% enzyme\ activity $$

V is inhibitor dependent rate of reaction while, Vmax is inhibitor independent rate of reaction.

### DPPH (2, 2-diphenyl, 1, picrylhydrazyl) scavenging assay

Brand-Williams assay [34] was used to find out DPPH free radical scavenging activity of isolated compounds of *E. umbellata*. About 24 mg, DPPH was dissolved in 100 mL methanol. Compounds solutions (1 mg/mL) were also prepared in methanol. Working solutions were prepared using serial dilutions in the concentrations range of 1000, 500, 250, 125, 62.5 and 31.05 μg/mL. About 0.1 mL of each working dilution was mixed with DPPH (3.0 mL) and incubated at 25 °C for 30 min. Absorbance was measured at 517 nm via UV-spectrophotometer (Thermo Electron Corporation: USA). Ascorbic acid was used as a standard. Results are presented as Mean ± SEM that has been calculated using the formula:

4 $$ \% DPPH\ Scavenging\ potential\kern0.5em =\kern0.5em \frac{\mathrm{standard}\ \mathrm{absorbance}\hbox{-} \mathrm{sample}\ \mathrm{absorbance}}{\mathrm{standard}\ \mathrm{absorbance}}\kern0.5em \times \kern0.5em 100 $$

### ABTS (2, 2′-Azino-bis (3-ethylbenzothiazoline-6-sulfonic acid) scavenging assay

According to the previously reported Re et al. [35] method antioxidant potential of isolated compounds of *E. umbellata* were determined against ABTS free radicals. ABTS (7 mM) and potassium persulfate (2.45 mM) solutions were mixed thoroughly and were incubated overnight in dark for the production of ABTS free radical. The absorption of this mixture was adjusted to 0.7 at 745 nm by adding methanol. About 300 μL compounds working dilutions and 3.0 mL ABTS solutions were mixed and incubated for 6 min. Finally the absorbance was measured via UV spectrophotometer. Ascorbic acid was used as positive control. % ABTS scavenging potential was measured using eq. 4.

### Animals

A total of 144 healthy Swiss male albino mice (25–30 g body weight) were obtained from National Institute of Health Islamabad Pakistan. All the animals were maintained in the animal house of the Department of Pharmacy, University of Malakand. The mice were housed in groups of six mice each in individual cages. All the animals were provided water and normal pellet diet and were kept under normal laboratory conditions with 12 h of light-dark cycle. All the animal procedures were conducted according to the UK animal scientific procedure act (1986) and approval was taken from the Departmental Animal Ethical Committee (DAEC/PHARM/2019/1).

### Acute toxicity studies of the CHF. Ext fraction and isolated compound-I

All animals were then treated orally with different doses of CHF. Ext (100, 200, 400, 500, 1000, 1500 and 2000 mg/kg body weight) and compound-**I** at doses of 50, 100, 150, 200, 250, 300 mg/kg body weight. Immediately after dosing, the mice were observed continuously for 4 h for symptoms of toxicity such as motor activity, convulsions, muscle spasm, tremors, sedation, lacrimation, hypnosis, diarrhoea, salivation, and loss of righting reflex. Mice were then kept under observation up to 72 h for any mortality. The CHF. Ext remained safe and nontoxic up to 2000 mg/kg and compound-**I** up to 300 mg/kg body weight dose range. Therefore, according to OECD guidelines, CHF. Ext at dose 200 mg/kg body weight that was 1/10th of 2000 mg/kg and compound-**I** at dose 30 mg/kg body weight that was 1/10th of 300 mg/Kg dose (maximum tested dose) was selected to evaluate the in-vivo anti-amnesic effect [36].

### Experimental design of inducing amnesia in mice

After the acclimatization period, in scopolamine-induced amnesia test the Albino male mice were randomly divided into six groups (*n* = 6) to study the effect of CHF. Ext and seven groups (n = 6) for compound-**I** (CGA). The doses of tested solutions of CHF. Ext have been presented in Table 1 while that of compound-**I** in Table 2. The treatments were continued for 8 days. In the Y-maze and Novel object recognition tests, scopolamine (1 mg/kg, i.p.) was given to the different groups only on the 8th day, 30 min after the respective treatments, to induce cognitive deficit in mice. One hour after administration of the drugs all animals were subjected to Y-maze and Novel object recognition test following standard procedure [10].

**Table 1:** Experimental design for CHF. Ext tretament groups used in the study

Group	Group Category	Treatment given	Route
I	Normal control	Normal saline (8 mg/kg)	p.o.
II	Negative control	scopolamine (1 mg/kg)	i.p.
III	Positive control	Donepezil (2 mg/kg) + scopolamine (1 mg/kg)	p.o, i.p
IV	Treatment group	CHF. Ext (50 mg/kg) + scopolamine (1 mg/kg)	p.o
V	Treatment group	CHF. Ext (100 mg/kg) + scopolamine (1 mg/kg)	p.o
VI	Treatment group	CHF.Ext (200 mg/kg) + scopolamine (1 mg/kg)	p.o

CHF.Ext, Chloroform fraction; p.o., Per oral; i.p., Intraperitoneal

**Table 2:** Experimental design for compound-**I** (CGA) tretament groups used in the study

Group	Group Category	Treatment given	Route
I	Normal control	Normal saline (8 mL/kg)	p.o.
II	Negative control	Scopolamine (1 mg/kg)	i.p.
III	Positive control	Donepezil (2 mg/kg) + scopolamine (1 mg/kg)	p.o. i.p.
IV	Treatment group	CGA (1 mg/kg) + Scopolamine (1 mg/kg)	p.o
V	Treatment group	CGA (3 mg/kg) + scopolamine (1 mg/kg)	p.o
VI	Treatment group	CGA (10 mg/kg) + scopolamine (1 mg/kg)	p.o

CGA, Chlorogenic acid; p.o., Per oral; i.p., Intraperitoneal

### Y-maze test for spontaneous alternation

The Y-Maze test was designed to evaluate the short term memory in experimental mice using reported procedure [37]. Y-maze apparatus is Y shaped with three arms of equivalent size, labelled as A, B, and C. Each arm was 20 cm long, 6 cm wide and 15.5 cm high and was oriented at an angle of 120° from the other two. For each animal the Y maze testing was carried out for 5 min. Mice were placed in one arm, and the order and number of arm entries were recorded. An arm entry was considered to be complete when the hind paws of the mice was completely inside a given arm while alternation was defined as consecutive entries by a mouse into the three different arms. The arena was cleaned using 70% v/v ethanol between each trial to avoid olfactory cues. The escape latency (second) on day 1–5 were recorded for all animals. At the end of 5th day, the spontaneous alteration behavior study was conducted. In spontaneous alteration study, each animal was placed at the center of the Y-Maze apparatus and allowed to move freely through the maze. The series of arm entries were visually recorded. To start behavioral test the mice was set in one arm and recorded the number of arm entries, same arm returns (SAR), and alternate arm returns (AAR) were measured. Percent spontaneous alternation performance (SAP) was calculated by the following formula:

5 $$ \% SAP\kern0.5em =\kern0.5em \frac{\mathrm{Active}\ \mathrm{alterations}\ \left(\mathrm{total}\ \mathrm{alterations}\right)}{\mathrm{Possible}\ \mathrm{alterations}\ \left(\mathrm{total}\ \mathrm{arm}\ \mathrm{entries}\right)}\kern0.5em -\kern0.5em 2x100 $$

### Novel object recognition test

The detail methodology [36] published, was followed while performing the novel object recognition (NORT) test. NORT consisting of habituation, sample, and test phases. On the day before test, mice were allowed to explore the box (without any object) for 2 min. On the day of test after habituation the sample phase was carried out 60 min after the last treatment on the 8th day. In sample phase each mouse was placed in an open field with two identical objects (plastic ball) for 5 min. The mouse was then returned to its home cage. The apparatus was comprised of a white coloured plywood box (40 cm × 40 cm × 66 cm) with a network floors that could be effortlessly cleaned (with 70%v/v ethanol) after every trial. The apparatus was illuminated by a 60 W light suspended 50 cm over the crate. The arena and objects were cleaned with 70% v/v ethanol between trials to avoid olfactory cues. For short-term memory, the test phase was performed 5 min after the sample phase. In the test phase, each mouse was placed again in the open field in which one of the identical objects had been replaced with a novel object (plastic square). The location of the object was counterbalanced so that one half of the mice in each group saw the novel object on the left side of the box arena, and the other half saw the novel object on the right side of the box arena to eliminate bias of sides. NORT can also be used for encoding and retrieval of spatial memory (long-term memory). A wash-out period of 5 days was allowed before the NORT to assess long-term memory in mice [38]. The procedure was the same as that for short-term memory except that mice were presented in the test phase 24 h after the sample phase exposure. The time spent exploring each object in each phase was recorded manually using a stopwatch. A mouse was scored as exploring when its head was oriented towards the object within a distance of 2 cm or when the nose was in contact with the object. Parameters including the time (in seconds) spent exploring familiar (F) object, time (in seconds) spent exploring the novel (N) object, and total time (in seconds) spent exploring both objects (N + F) were measured separately. Percentage of discrimination index (DI) was calculated by formula:

6 $$ \% DI\kern0.5em =\kern0.5em \frac{\mathrm{N}\hbox{-} \mathrm{F}}{\mathrm{N}+\mathrm{F}}\kern0.5em \times \kern0.5em 100 $$

### Isolation of frontal cortex and hippocampus

Immediately after the Y-Maze test and NORT, all the animals were sacrificed by cervical dislocation before decapitation to provide each animal with a quick and painless death using previous procedure illustrated in schedule-1 of UK, animal scientific procedure act 1986. The frontal cortex (FC) and hippocampus (HC) were dissected out in ice cold 0.1 M phosphate buffer saline (pH 8.0). The tissues were weighed and 20 mg tissue/mL homogenate of brain samples was prepared in phosphate buffer (pH 8.0). The homogenates were centrifuged at 10,000 rpm for 10 min at 4 °C, and the resulting supernatant was used for the estimation of anti-cholinesterase activity following Ellman’s [33] and antioxidants assays [34, 35].

### Molecular docking validation for anti-amnesic activity

For theoretical study towards anticholinesterase activity, two dimensional structure of molecules were drawn on Chem Draw Professional 16.0 (PerkinElmer Inc.) and finally adapted to 3D conformations followed by energy minimizations using Chimera 1.13.1rc. Docking simulations were performed on GOLD (Genetic Optimizations for Ligands) software (GOLD suit 5.6.3, Cambridge Crystallographic Data Center) [39]. Gold score was selected as a fitness function for the ligand molecules. A search area of 6 Å radius was fixed for docking simulations in the active sites of the reference ligands. Acetyl cholinesterase and butyryl cholinesterase (PDB ID: 4BDS) were selected as receptor enzymes with well-established crystal structures recovered from the website of Protein Data Bank (www.rcsb.org). These structures of protein were subsequently prepared by hydrogen addition and water and co-crystallized ligands elimination. Default settings were adopted for all parameters. Areas where reference ligands were bound to enzymes were designated as active sites. The HPLC detected and isolated compounds were docked into designated active gorges to see their possible interactions with different amino acids. Docking accuracy was also validated by redocking of the reference ligands. The different images (2D & 3D) were visualized and processed by using Discovery Studio Visualizer software [40].

### Statistical analysis

All in-vitro experiments were performed in three replicates by applying two way ANOVA followed by Bonferroni Post-test to determine the values of P. The results were represented as Mean with SEM.

For in vivo analysis Student’s t-test, one way ANOVA followed by Dunnett’s posthoc multiple comparisons & two way ANOVA followed by Bonferroni Post-test were used to determine the values of P. *P* < 0.05 were considered as significant. Different animals groups: Scopolamine treated group, Donepezil treated group (standard control), Normal saline treated group, CGA & CHF. Ext treated group (test control) were used in the in vivo study. Values were significantly different in comparison to scopolamine treated group. ***: *p* < 0.001, **: *p* < 0.01,*: *p* < 0.05 and *ns*: Values not significantly different in comparison to Scopolamine treated group.

### Assessment of IC_50_ values

Linear regression was used to calculate IC_50_ from % inhibition of data of AChE and BChE by different concentration of test samples using Excel program 2007.

### Regression (*y*) and linear correlation (R^2^)

Regression (*y*) and linear correlation (R^2^) were used to determine TPC, TFC, the antioxidant and enzyme inhibition potentials of samples using Excel 2007.

## Results

### Preliminary screening of phytochemicals

*E. umbellata* fruit Met. Ext give positive results for preliminary major phytochemical groups that are presented in Table 3.

**Table 3:** Preliminary qualitative phytochemical screening of Met. Ext from *Elaeagnus umbellata* Thunb. fruit

S. No	Phytochemical groups	Reagents used	Analyses	Results
**1**	Alkaloids	Dragendorff’s	Orange red color precipitate	**+**
**2**	Flavonoids	Ferric-chloride	Yellow color and after HCL addition becomes colorless	**+**
**3**	Glycosides	Keller Killiani	Formation of red to brown layer	**+**
**4**	Tannins	Gelatin	Brownish-green precipitate	**+**
**5**	Triterpenoids	Liebermann Burchard	Reddish brown boundary	**+**
**6**	Anthraquinones	Bontrager’s	Formation of reddish color	**+**

### Total phenolic content (TPC)

**Fig. 1:**
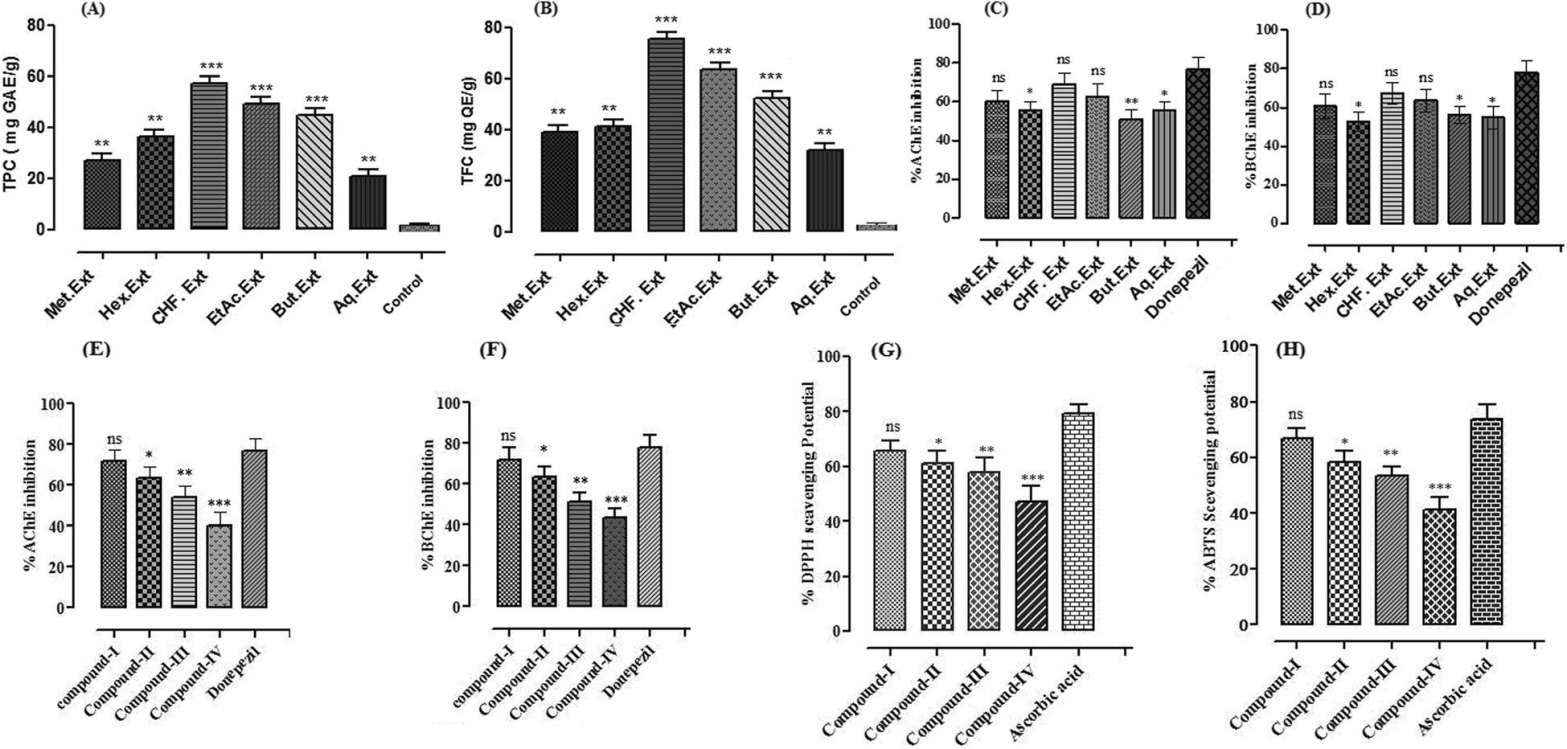
Total phenolic/flavonoids contents, anticholinesterase and antioxidant potential of extract/fractions and isolated compound of *Elaeagnus umbellata* Fruit. {(**a**) Total phenolic content expressed as gallic acid equivalents (mg GAE)/g dry plant sample; (**b**) Total flavonoid content expressed as quercetin equivalents (mg QE)/g dry plant sample; (**c**) %AChE and (**d**) %BChE inhibition potential of extract/fractions; (**e**) %AChE and (**f**) %BChE inhibition potential of isolated compounds; (**g**) DPPH and (**h**) ABTS free radical scavenging activity of isolated compounds}

Standard Gallic acid curve was constructed by preparing the dilutions 20, 40, 60, 80 and 100 mg/mL to estimate the TPC in *E. umbellata* fruit samples using graphical regression method (Fig. 1a). Comparatively higher TPC contents were estimated in almost all fractions than the control sample (1.25 ± 0.55 mg GAE / g of dry sample) (Table S1). CHF. Ext and EtAc. Ext fractions (56.97 ± 0.77 and 49.15 ± 1.05 mg GAE / g of dry sample) showed highest percentage of total phenolic contents amongst the samples.

### Total flavonoid contents (TFC)

To estimate the TFC in *E. umbellata* fruit hydro methanolic extract and various fractions, a regression curve of standard quercetin was constructed by preparing the dilutions 20, 40, 60, 80 and 100 mg/mL and the regression equation was used for TFC estimation. The estimated contents are graphically presented in Fig. 1b. Highest total flavonoid content was present in CHF. Ext and EtAc. Ext fractions (75.53 ± 0.44 and 63.49 ± 1.03 mg QE/g of dry sample respectively; Table S1). The results are statistically significant in comparison with the control (1.59 ± 0.25 mg QE/g of dry extract sample).

### In vitro cholinesterase inhibitory potential of *E. umbellata* Thunb. Fruit extract/fractions and isolated compounds

Cholinesterase (AChE and BChE) inhibition by *E. umbellata* Thunb. Extract/fractions were determined at various concentrations and are presented in Figs. 1c to f (Table S2). Among different fractions, CHF. Ext and EtAc. Ext showed highest percent activities against AChE (87 ± 1.2 and 84 ± 1.0) with IC_50_ values of 33 and 55 μg/mL respectively (Fig. 1c). Similarly, CHF. Ext and EtAc. Ext fractions also showed highest percent inhibition against, BChE which were 86 ± 0.3 and 82 ± 0.5 with IC_50_ values 35 and 58 μg/mL respectively (Fig. 1d). Methanolic extract and subsequent fractions showed concentration dependent activities. Donepezil have been used as positive control that showed % inhibition of 92 ± 0.3 with IC_50_ of 28 μg/mL.

Similarly, the %AChE and %BChE inhibition potential of isolated compounds (**I-IV**) are presented in Fig. 1e & f (Table S3). Compound **I** showed prominent inhibition against AChE (90 ± 0.2) with IC_50_ value 31 μg/mL. While, compound **III**, **II** and **IV** showed % inhibition of 76 ± 2.1, 81 ± 0.5, and 66 ± 1.2 μg/mL with IC_50_ values 45, 59 and 92 μg/mL respectively (Fig. 1e). Interestingly the isolated compounds **I** and **II** showed comparable results with the standard drug donepezil that showed % inhibition of 93 ± 0.5 against AChE with IC_50_ value 25 μg/mL.

Similarly, these compounds also showed high percent inhibition of BChE as well. Compound-**I** showed highest % inhibition of 88 ± 0.2 with IC_50_ of 32 μg/mL (Table S3). Other compounds like **II**, **III**, and **IV** showed % inhibition of 80 ± 0.5, 66 ± 1.5, and 58 ± 2.2 with IC_50_ values 62, 83, and 115 μg/mL respectively (Fig. 1f). All the compound showed concentration dependent inhibitory activity.

### In vitro DPPH and ABTS scavenging potential of isolated compounds

DPPH and ABTS inhibitory potential of compounds **I-IV** are presented in Fig. 1g & h. The results indicate that compound-**I** causes highest % inhibition which was comparable to standard ascorbic acid. The results indicates that compound-**I**, causes significant % inhibition potential with lowest IC_50_ values 35 and 38 μg/mL against DPPH and ABTS (Table S4).

### Relationship of total phenolic/flavonoid contents of CHF. Ext versus cholinesterase inhibitory activity

Highest value of regression coefficient was obtained for AChE (R^2^ = 0.988) and BChE (R^2^ = 0.9779) inhibition when plotted against total phenolic contents of CHF. Ext (Figure S1B). Similarly, the correlation co-efficient for AChE and BChE inhibition verses total flavonoid contents were found to be 0.9301 and 0.9928 respectively (Figure S1D).

### Sstructural confirmation and characterization of isolated compounds

#### Compound-I: Chlorogenic acid

Compound-**I** was isolated as amorphous colourless solid with molecular formula C_16_H_18_O_9_ on the base of its FAB-MS ion peak at m/z 353.3. The R_*f*_ value of compound-**I** is 0.45 in solvent system of n-hexane and ethyl acetate (3:7) while the melting point was 208–209 °C and was soluble in methanol. The structural formula of compound-**I** is presented in Figure S2. The FTIR spectrum of compound-**I** is presented in Figure S3. Peak at 3647.39 cm^− 1^ demonstrating the -OH group while C-H bond stretching occurs at 3072.60 cm^− 1^ peak. Aromatic nucleus has been detected from C=C strong absorption bands at 1687.71 cm^− 1^. The carbonyl functionality (C=O) occurs at 1639.49 cm^− 1^. The broad peak at 3329.14 cm-^1^ representing the carboxyl group. The FAB-MS is represented in Figure S4. Compound-**I**^1^H-NMR spectrum indicated singlet’s at 4.18 (dt, J = 5.2, 3.3 Hz, 1H), 3.74 (dd, J = 8.6, 3.2 Hz, 1H), 5.34 (ddd, J = 9.6, 8.5, 4.4 Hz, 1H), 7.05 (d, J = 2.1 Hz, 1H), 6.78 (d, J = 8.1 Hz, 1H), 6.95 (dd, J = 8.2, 2.1 Hz, 1H), 7.56 (d, J = 15.9 Hz, 1H) and 6.26 (d, J = 15.9 Hz, 1H), corresponding to the H-3, H-4, H-5, H-2′, H-5′, H-6′, H-7′ and H-8′ position, while the two CH_2_ protons (α and β) were assigns multiplets at δ 2.04 and 2.18 at position 2 and δ 2.24 and 2.07 at position 6, respectively presented in Figure S5.

The ^13^C-NMR spectrum showed 16 signals, including eight methines, six quaternary and two CH_2_ carbons (Figure S6). ^13^C NMR (126 MHz, MeOD) spectral data δ 76.11, 38.13, 71.27, 73.45, 71.89, 38.75, 176.99, 127.73, 115.19, 146.68, 149.46, 116.45, 122.97, 147.04, 115.17, 168.65, 48.98 are presented in Table S5.

#### Compound-II: Ellagic acid

The compound-**II** was isolated as tan to gray colour powder in solvent system n-hexane and ethyl acetate (2:8) with molecular formula C_14_H_6_O_8_ on the base of its EIMS ion peak at m/z302.197 g/mol. The R_*f*_ value of compound-**II** is 0.57 while the melting point is ≥350 °C while boiling point is 364 °C. The structural formula of compound-**II** is presented in Figure S2. ^1^H NMR (Chloroform-d,500 MHz): δ = 7.27 (s, 2 H) the chemical shift of δ = 7.27 most probably represents the 2 protons attached on position 9 and 21 of the given structure, δ = 1.56 ppm (s, 2 H), the peak on the high chemical shift of 1.56 might be hydroxyl group protons at position 6 and 18 of the given structure, 0.08 ppm (s, 2H) the singlet peak on very high chemical shift of 0.08 represents the proton of –OH group on position 8 and 20 of the given structure (Figure S7). ^13^C NMR (Chloroform-d, 100 MHz): δ = 108, 51.8, 48, 42, 37.6, 13.7 ppm (Figure S8). The EIMS of compound-**II** is given in Figure S9.

#### Compound-III: Gallic acid

The compound-**III** was isolated as Light yellowish white crystalline powder in solvent system n-hexane and ethyl acetate (4:6) with molecular formula C_7_H_6_O_5_ on the base of its EIMS ion peak at m/z170 g/mol. The melting point of the compound is 260 °C. The compound-**III** is soluble in ethanol, methanol, ether, glycerol, ethyl acetate and acetone with R_*f*_ value 0.35. The structural formula of compound-**III** is given in Figure S2. ^1^H NMR (Methanol-d_4_, 500 MHz): The proton NMR spectrum of the given compound has been provided in Figure S10. The exploited solvent was Methanol-d, which has been detected at 3.35 ppm as given in the spectrum. δ = 7.08 (s, 2 H) this singlet peak represents 2 protons attached on carbon number 3 and 10 of the given structure, δ = 4.93 ppm (br. s., 3 H) this broad singlet peak might be the proton attached as –OH group at carbon number 5, 7 and 9 of the identified structure. ^13^C NMR (Methanol-d_4_, 100 MHz): δ = 169.0, 145.0, 138.2, 120.6, 108.9 ppm (Figure S11). The EIMS is represented in Figure S12.

#### Compound-IV: Phloroglucinol

The compound-**IV** was isolated as colourless to beige solid in solvent system n-hexane and Ethyl acetate (6:4) with molecular formula C_6_H_6_O_3_ on the basis of its EIMS ion peak at m/z 126.1. The melting point of the compound is − 219 °C and R_*f*_ value is 0.39. The compound is soluble in methanol, ethanol, and diethyl ether. The structural formula of compound-**IV** is given in Figure S2. The FTIR spectrum of compound-**IV** is presented in Figure S13. The absorption peaks at 3161.33 and 3479.58 cm^− 1^ demonstrating the –OH group. Peak at 2887.44 cm^− 1^ indicates C-H stretching. Aromatic nucleus can be recognized by C=C strong peak at 1612.49 cm^− 1^. Band at 1739.79 cm^− 1^ is due to functionality of 1, 3, 5 substituted benzene.

The NMR signals of compound-**IV** in methanol solvent is reported below: 1H NMR (MeOD, 300 MHz) δ 9.11 (s, 11H), 5.83 (s, 3H, CH) (Figure S14, Table S6) while 13C NMR (MeOD, 75 MHz) δ160.0 ppm (CH2), 95.5 ppm (CH) (Figure S15, Table S6). The EIMS is shown in Figure S16.

### In vivo antiamnesic potential of extract/fractions and isolated compounds

#### Effects of extract/fractions and isolated compounds in Y-maze test

**Fig. 2:**
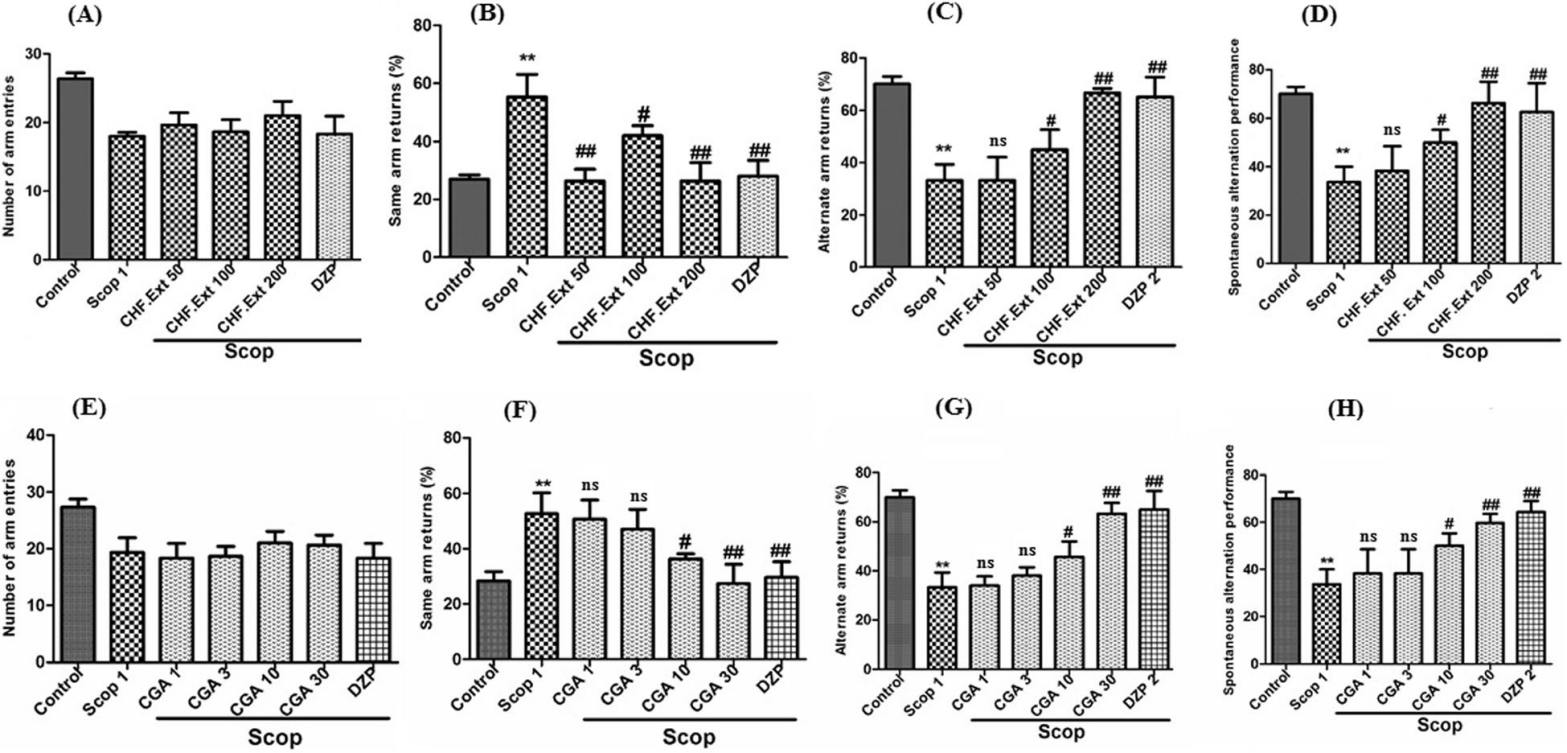
Effect of CHF. Ext and isolated compound chlorogenic acid of *Elaeagnus umbellata* Thunb. on mice in the behavioural Y-maze test. {(**a**) number of arm entries (**b**) Same arm returns (**c**) alternate arm returns (**d**) % Spontaneous alteration performance were recorded in CHF. Ext (50, 100 and 200 mg/kg) groups versus scopolamine (Scop. 1 mg/kg) treated group; (**e**) number of arm entries (**f**) Same arm returns (**g**) alternate arm returns (**h**) % Spontaneous alteration performance were recorded in isolated compound chlorogenic acid (1, 3, 10 and 30 mg/kg) treated groups versus scopolamine (Scop. 1 mg/kg) treated group}

Y maze test results are presented in Fig. 2. A non-significant (*p* > 0.05) decrease was observed in the total numbers of entries into arms of instrument both in scopolamine and treated groups (CHF.Ext and CGA) when compared to control group (Fig. 2a & e). Returns to the same arms were significantly (*p* < 0.01) high in group treated with scopolamine in comparison to control group. CHF. Ext (200 mg/kg body weight), isolated compound CGA (10 and 30 mg/kg body weight) and donepezil (2 mg/kg) showed significant (p < 0.01) reduction in the percentage of same arm returns comparable to scopolamine treated group (Fig. 2b & f).

The CHF. Ext (200 mg/kg), isolated compound CGA (10 and 30 mg/kg body weight) and standard drug donepezil (2 mg/kg body weight) groups contrarily showed a significant (*p<0.05; **p < 0.01) increase in the percentage of alternate arm returns as compared to scopolamine treated group where a significant decrease in the number of alternate arm returns was observed (*p*<0.01; Fig. 2c & g). At the end of 5th day of experimental cycle, spontaneous alteration behavior study was conducted (Fig. 2d & h). In spontaneous alteration study, each animal was placed at the center of the Y-Maze apparatus and allowed to move freely through the maze in three trials of 8-min session each. The series of arm entries were visually recorded. Dose dependent increase was also observed in % spontaneous alteration performance (Fig. 2d & h) which is significant (*p* < 0.01) for CHF. Ext (200 mg/kg) and comparable to the donepezil (2 mg/kg body weight). Results of Y-maze test conducted to determine escape latency (seconds) are given in Figure S17. Scopolamine treated group shown highest escape latency from day 1–5. The high escape latency time indicates poor cognitive function. Donepezil (standard control), normal control group, and treated groups (CHF.Ext & CGA) were observed with significant decline (*p* < 0.001)*** in escape latency over the whole experimental cycle that pointed towards the improved memory of the treated animals over the diseased control group.

#### Effects of active extract/fractions and isolated compounds in novel object recognition test

**Fig. 3:**
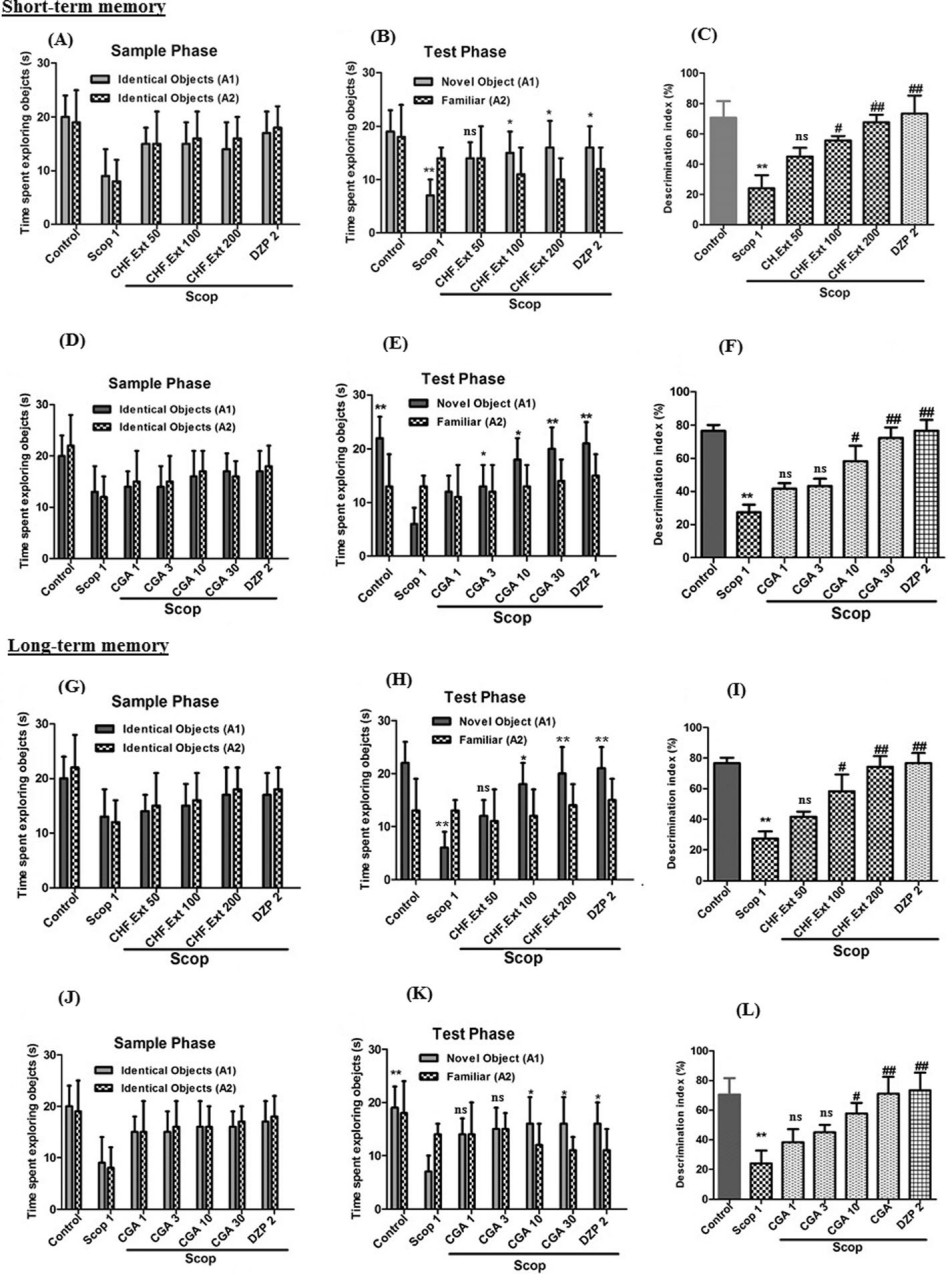
Effect of CHF. Ext and isolated compound (CGA) of *E. umbellata* Thunb. on mice in behavioural NORT {(**a**) Time spent in the sample phase (**b**) Time spent in the test phase (**c**) % Discrimination index were recorded in CHF. Ext (50, 100 and 200 mg/kg) treated groups versus scopolamine (Scop. 1 mg/kg) treated group and (**d**) Time spent in the sample phase (**e**) Time spent in the test phase (**f**) % Discrimination index were recorded in isolated compound chlorogenic acid (CGA: 1, 3, 10 and 30 mg/kg) treated groups versus scopolamine (Scop. 1 mg/kg) treated group for assessment of short-term memory in mice model in behavioral NORT; (**g**) Time spent in the sample phase (**h**) Time spent in the test phase (**i**) % Discrimination index were recorded in CHF. Ext (50, 100 and 200 mg/kg) treated groups versus scopolamine (Scop. 1 mg/kg) treated group and (**j**) Time spent in the sample phase (**k**) Time spent in the test phase (**l**) % Discrimination index were recorded for isolated compound chlorogenic acid (1, 3, 10 and 30 mg/kg) treated groups versus scopolamine (Scop. 1 mg/kg) treated group for assessment of long-term memory in mice model in behavioral NORT}

The results obtained with the novel object recognition test are shown in Fig. 3. To assess short term memory in the sample phase indicates that no significant difference in consuming time for exploration of two objects between CHF. Ext (Fig. 3a), CGA (Fig. 3d) treated groups, and scopolamine treated group. However, CHF. Ext (50, 100 and 200 mg/kg), CGA (10 and 30 mg/Kg) and donepezil (2 mg/kg) groups consume more time with novel object in the test phase as compared to scopolamine treated group (*p < 0.05, **p < 0.01). Also, scopolamine (1 mg/kg) treated group spent more time with familiar object compared to novel object (*p* < 0.01) (Fig. 3b & e). The % Discrimination index (DI) was significantly high for CHF. Ext (50, 100 and 200 mg/kg), CGA (10 and 30 mg/Kg), and donepezil (*p < 0.05, **p < 0.01) groups when compared to scopolamine treated group. All groups show % DI above 50% while scopolamine group has shown significantly low value as compared to control (*p* < 0.01) (Fig. 3c & f). Similarly, in the sample phase of long term memory task, no significant differences were observed in the total time spent exploring the two identical objects between the CGA, CHF. Ext and scopolamine treated group (Fig. 3g & j). A significant (*p < 0.05, **p < 0.01) increase occurs in the test phase of long term memory in spending the time for exploration of novel object with CHF. Ext, (100 & 200 mg/kg), CGA (10 & 30 mg/Kg) and donepezil (2 mg/kg) treated groups. However, scopolamine group has shown a significant (*p* < 0.01) increase in exploration time with the familiar object compared to novel one (Fig. 3h & k). The CHF. Ext (100, 200 mg/kg), CGA (10 & 30 mg/Kg) and donepezil 2 mg/kg groups have shown significant (*p < 0.05, **p < 0.01) increase in %DI, while scopolamine 1 mg/kg group has significantly (*p* < 0.01) lower %DI as compared to control (Fig. 3 i & l).

#### Effect of isolated compounds and extract fraction on brain Cholinesterases (AChE and BChE) activity in mice

**Fig. 4:**
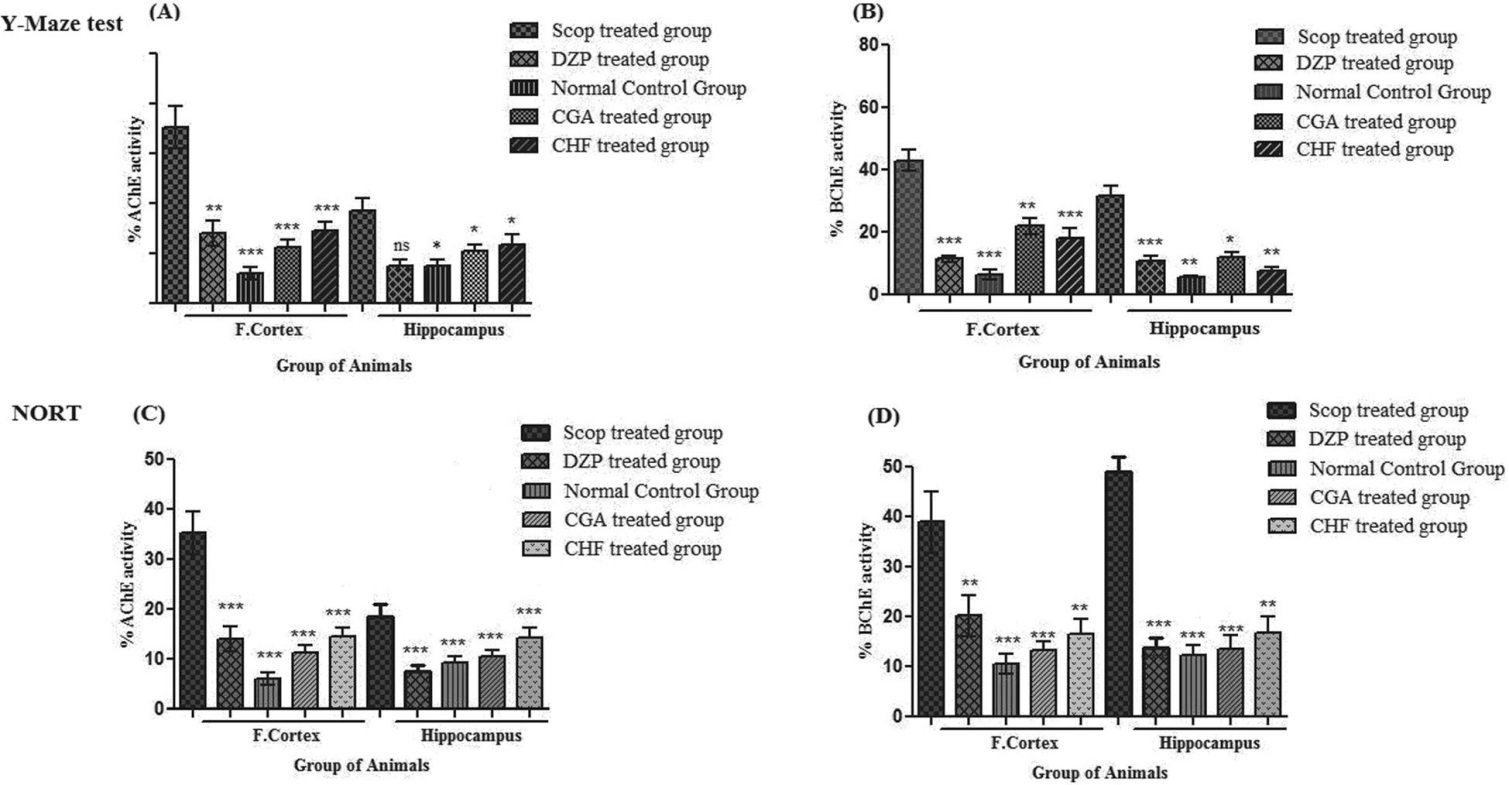
Ex-vivo % AChE and BChE activity in the frontal cortex and hippocampus of different animal groups {(**a** & **b**) Different animal groups Scopolamine (Scop. 1 mg/kg) treated group, Donepezil (DZP) treated group, Normal control group, CGA & CHF treated group were used in behavioral Y maze test to assess AChE and BChE activity; while (**c** & **d**) has shown AChE and BChE activity in NORT}

Ex vivo analysis of cholinesterase inhibitory potentials was also measured to check the cholinergic hypo function in animal model using the isolated compound CGA and CHF extract. One hour after administration of the last dose of isolated compound CGA (10 and 30 mg/kg; (i.p.) and CHF. Ext (200 mg/kg; (i.p.), animals were sacrificed. Frontal cortex and hippocampal tissues were isolated in ice cold phosphate buffer and supernatants were collected from homogenized part of brain tissues at a standardized protein content of 5 mg/ml. %AChE and %BChE activity results in frontal cortex and hippocampus of different groups of animals in Y-Maze test are given in Fig. 4. Significantly (***p < 0.001) high AChE and BChE activity was observed in the frontal cortex and hippocampus tissues of scopolamine treated group (Fig. 4a and b). A significant decline (**p < 0.01; ***p < 0.001) in %AChE and BChE activity was observed in Donepezil treated group (standard control group) in the cortex and hippocampus regions respectively. The %AChE activity in the cortex and hippocampus tissues of normal control group were significantly different (***p < 0.001; **p < 0.01) from scopolamine treated group.

%AChE activities in frontal cortex and hippocampus of different groups of animals in Novel object recognition test are given in Fig. 4c. Percent AChE and BChE activity in the cortex and hippocampus of scopolamine treated group was significantly (***p < 0.001) higher than that of the control group. %AChE (A) and BChE (Fig. 4c & d) activity in the cortex and hippocampus of Donepezil treated groups were significantly (***p < 0.001; **p < 0.01) lower as compared to scopolamine treated group. The results of % AChE and BChE activity of CGA and CHF treated groups in the cortex and hippocampus tissues were also significantly (***p < 0.001; **p < 0.01) lower when compared to scopolamine treated group.

#### Effect of isolated compounds and extract fraction on brain DPPH free radicals scavenging activity in mice

**Fig. 5:**
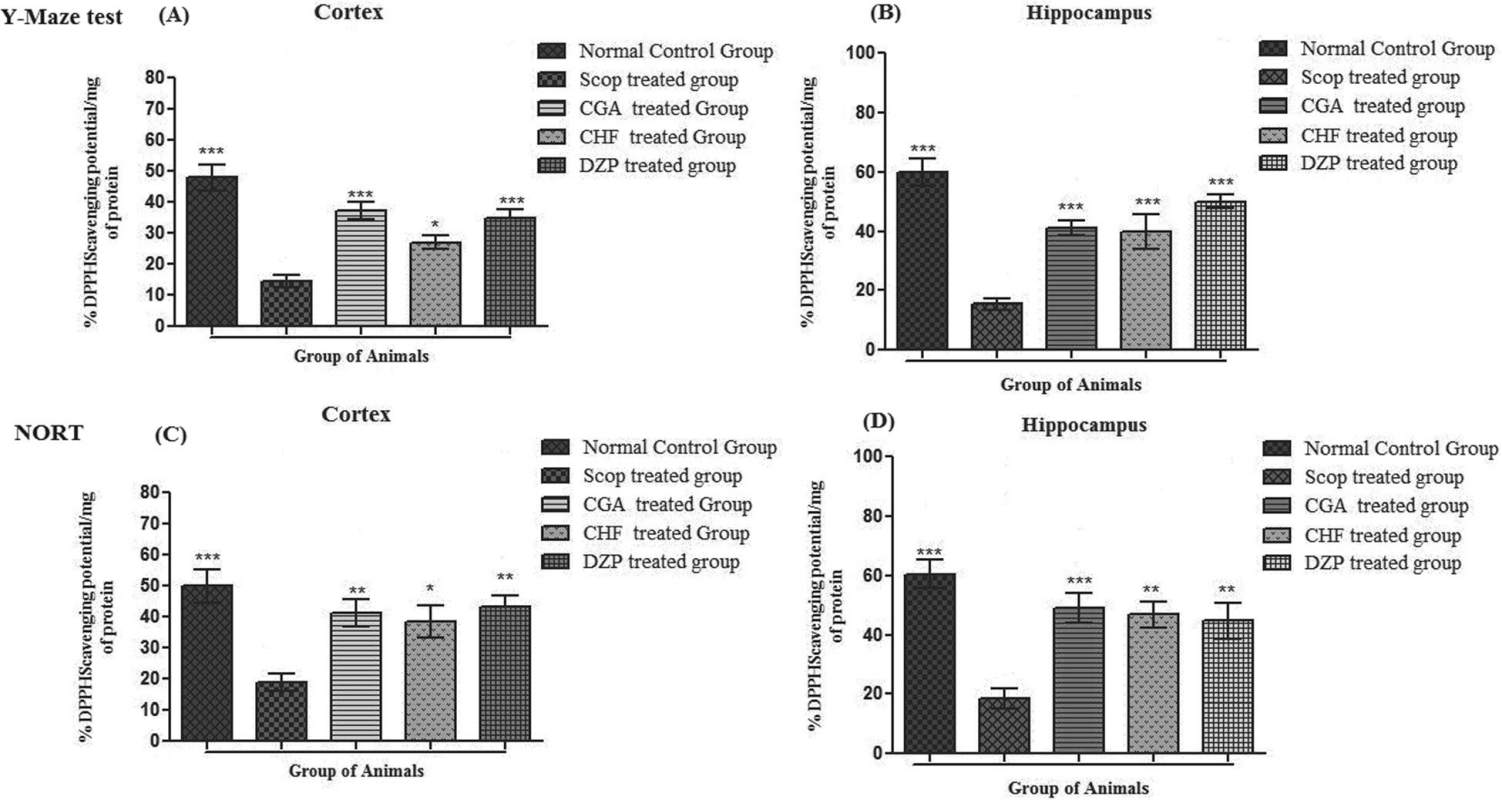
Ex-Vivo %DPPH free radical scavenging effects in frontal cortex and hippocampus of different animal groups {(**a** & **b**) Different animal groups (Scopolamine (Scop. 1 mg/kg) treated group, Donepezil (DZP) treated group, Normal control group, CGA & CHF treated group) were used in behavioral Y maze test to assess %DPPH inhibition potential in frontal cortex and hippocampus of different animal groups; while (**c** & **d**) has shown %DPPH inhibition in NORT}

Results regarding ex vivo %DPPH free radicals scavenging potential in cortex and hippocampus of different animal groups in Y-Maize test are summarized in Fig. 5a and b. % antiradicals activity of Normal control group was significantly high (***p < 0.001) than that of Scopolamine treated group. Furthermore, Donepezil treated and test control groups like CGA and CHF showed: 39.62 ± 4.12, 47.85 ± 2.11 and 30.53 ± 1.65% DPPH inhibition potential in cortex and 52.41 ± 1.43, 44.12 ± 1.77, 32.63 ± 2.47% in hippocampus respectively.

Ex vivo %DPPH free radicals scavenging potential in cortex and hippocampus of different animals groups in Novel object recognition test are summarized in Fig. 5c & d. Percent DPPH inhibition activity in the cortex and hippocampus of normal control group was significantly (***p < 0.001) higher than that of scopolamine treated group. Donepezil, CGA and CHF treated groups showed significantly higher % anti-radicals activities: 55.41 ± 4.55, 51.43 ± 2.88, 45.74 ± 2.47 in cortex (C) and 59.58 ± 4.11, 65.36 ± 1.45, 62.54 ± 3.52 in hippocampus (D) respectively as compared to standard group.

#### Molecular docking validation of isolated compounds for anticholinesterases (AChE & BChE)

**Fig. 6:**
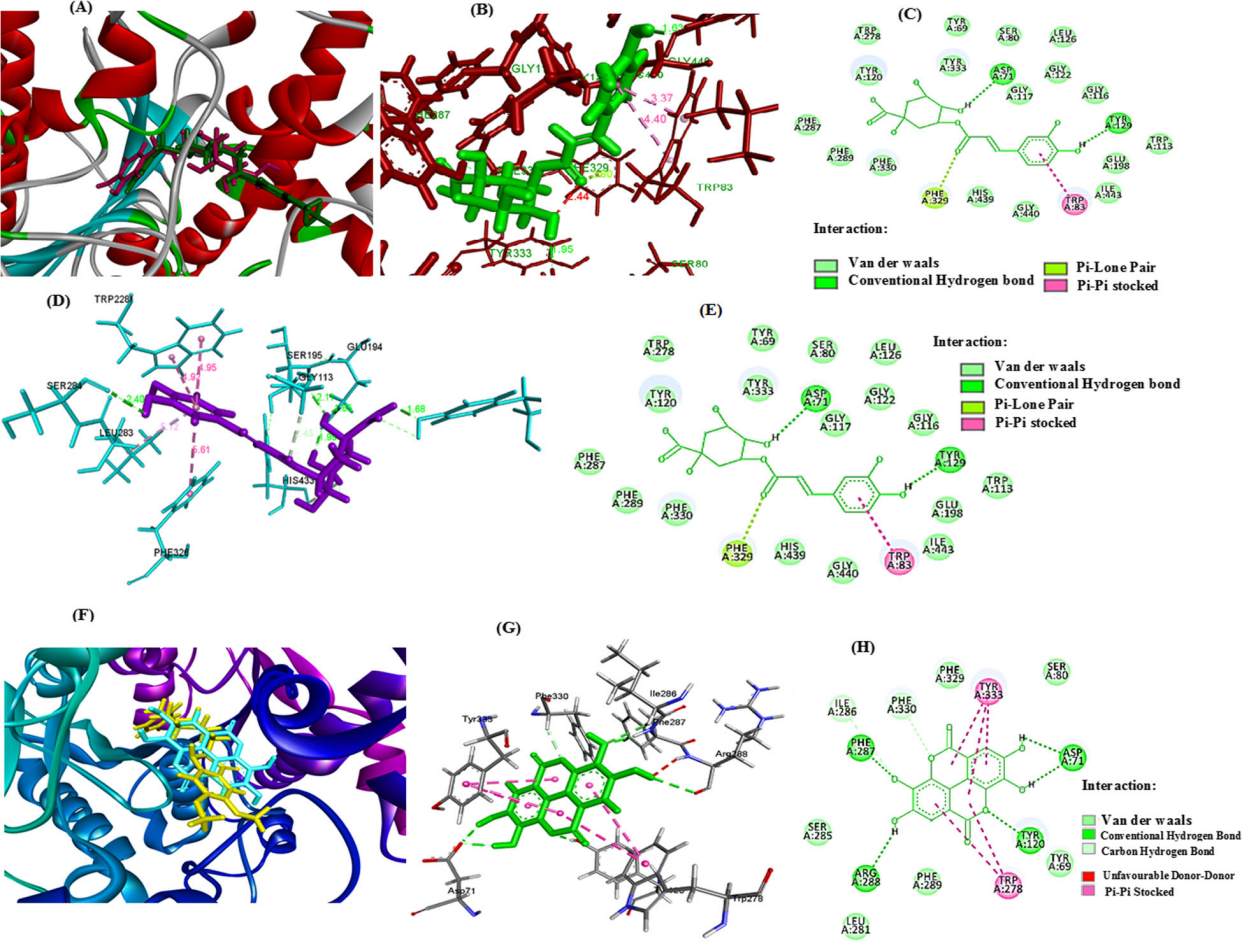
Molecular docking validation of compounds chlorogenic acid and ellagic acid against AChE & BChE {(**a**) Superimposed ribbon diagram for chlorogenic acid and donepezil (**b**) Stereo view of the docking posture of chlorogenic acid (green color stick model) in the binding pocket of AChE enzyme (**c**) 2D interactions of chlorogenic acid. **d** Stereo view of the docking posture of chlorogenic acid (purple color stick model) in the binding pocket of BChE enzyme (**e**) 2D interactions of chlorogenic acid (**f**) Superimposed ribbon diagram for ellagic acid and donepezil (**g**) Stereo view of the docking posture of ellagic acid (green color stick model) in the binding pocket of AChE enzyme (**h**) 2D interactions of ellagic acid}

To study the molecular docking of cholinesterase (AChE & BChE) inhibition; binding analyses of the isolated compounds were performed on a GOLD suit v5.6.3. For this purpose, crystal structures of *Tc*AChE in complex with donepezil from *Tetronarce californica* (PDB ID: 1EVE) and *h*BChE in complex with tacrine (PDB ID: 4BDS) from *Homo sapiens* were used as receptors. The docking pose of most active compound chlorogenic acid superimposed onto donepezil inside the binding cavity of *1EVE* is shown in Fig. 6a**.** Analysis of binding modes indicates similar binding orientations for chlorogenic acid and donepezil in the active gorge of the receptor protein. Upon visual inspection (Fig. 6b & c), we can observe interactions such as π-π stacking (TRP83 to phenolic ring), conventional hydrogen bonding (TYR129 to OH of phenolic ring), pi-lone pair (PHE329 to C=O group of caffeoyl moiety), and numerous van der Waals attractions for the best docking pose of chlorogenic acid (Gold fitness score = 62.69).

For studying the mechanism of inhibition for human BChE, the crystal structure with PDB ID of 4BDS was used as target protein. The mechanism of BChE inhibition by chlorogenic acid was studied by viewing its interactions in the active gorge of human BChE (PDB ID: 4BDS) as the enzyme model. The structural features of the most active compound chlorogenic acid suggest that it must have interactions like hydrophilic and hydrophobic in the active site of the enzyme. Upon visual inspection of the best docked pose (Fig. 5d & e), chlorogenic acid can be seen to bind hydrophobically (π-π stacking) with amino acid residues LEU283, PHE326, and TRP228. Numerous hydrogen bonding interactions between amino acids (TYR125, SER195, and GLU194) and hydroxyl groups of chlorogenic acid also seems to contribute to stability of chlorogenic acid-BChE complex.

The docking posture of the active ellagic acid superimposed onto donepezil in the inner surface of the binding cavity of *1EVE* is shown in Fig. 6f**.** Examination of different binding modes indicates similar binding coordination for ellagic acid and donepezil in the active pocket of the receptor protein. Upon visual inspection (Fig. 6g & h), different interactions can be seen such as π-π stacking (TRP278, TYR333), conventional hydrogen bonding (TYR120, ARG288, PHE287, ASP71 to OH of phenolic ring), carbon hydrogen bond interactions (PHE330, ILE286), unfavorable donor-donor interaction (ARG288 to OH group) and various number of van der Waals attractions for the best docking pose of ellagic acid (Gold fitness score = 55.24).

The Gold score values and energy of ligands of isolated compounds are presented in Table 4. The chlorogenic acid and ellagic acid have higher Gold score values with highest cholinesterase inhibitory activities while, gallic acid and phloroglucinol showed weaker inhibitory activity. The more Gold Score values represent tighter binders.

**Table 4:** The Gold scores and energy of ligand values of cholinesterase inhibitors present in *Elaeagnus umbellata* Thunb. fruit

Compound	Compound name	Gold Score	Energy of ligand
Compound-I	Chlorogenic acid	62.69	−10.556
Compound-II	Ellagic acid	55.24	−6.971
Compound-III	Gallic acid	39.42	−6.917
Compound-IV	Phloroglucinol	33.28	−5.071

The Chlorogenic acid and Ellagic acid have higher Gold score values with highest cholinesterase inhibitory activities while, Gallic acid and Phloroglucinol showed weaker inhibitory activity. The more Gold Score values represent tighter binders [40]

## Discussion

Memory is the process where experiences are documented in brain that can be used to acclimate their responses to the environment. It has great importance in some one life to define itself and their place along with normal working life. Central cholinergic system is considered to be the most important neurotransmitter involved in regulation of cognitive functions. Acetyl cholinesterase is the key enzyme responsible for acetylcholine hydrolysis which terminates the cholinergic transmission. Decrease in the cholinergic conduction is associated with cognitive dysfunction and are reported in neurodegenerative diseases such as Alzheimer’s disease (AD). Literature studies have revealed that cholinesterase inhibitors might act on several therapeutic targets such as AChE enzyme that is responsible for acetylcholine hydrolysis [41, 42]. However, there is still a need of novel AChE inhibitors with low toxicity and high penetration rate to the central nervous system. The dementing illness that has received the most attention in the past decade is AD, while impaired cognitive functions are the major features of AD. The only standard drugs used as acetyl cholinesterase inhibitors for AD are galantamine, donepezil and rivastigmine [41]. Natural products have already demonstrated to be promising sources of functional acetyl cholinesterase (AChE) inhibitors. The standard drugs for AD treatment, galantamine and rivastigmine are alkaloids derived from plants [42]. AChE inhibition is also considered as a remedial strategy for other types of neuronal disorders like dementia and Parkinson’s diseases [43]. There is still a need to explore new plants for new potent and enduring AChE inhibitors with low or no side effects. Many plant species from various parts of the world have been evaluated for anti-cholinesterase activity [44, 45].

It is obvious of our current investigational study that *E. umbellata* extracts displayed various bioactive constituents including alkaloids, flavonoids, glycosides, tannins, terpenoids, anthraquinones and pigments provide a significant source of secondary metabolites which play a role as cholinesterase inhibitors [21, 22, 46]. In the present study, both the chloroform extract and isolated compounds were found potent to inhibit the Acetyl cholinesterase enzyme and restoring the cholinergic functions. This property tends to allow more retention of acetylcholine in the brain, which is important for the cognitive function, learning and memory. The present study demonstrates the beneficial effect of chloroform extract and chlorogenic acid of *E. umbellata* on scopolamine-induced amnesia. Oral suspension of the extracts and isolated compound was fed to the animals used in rodent models of memory and learning, viz. Y maze and Novel object recognition test. Behavioural study was further confirmed by estimation of the brain biochemical such as brain cholinesterases (AChE and BChE) activity and DPPH free radicals scavenging activity in mice. Y maze spontaneous alteration test is used for studying working memory and exploration of the animals. The test is based on the willing of the animals to explore new environment. The normal animals will explore the new arm, the animals whose memory is not working properly will again enter the old arm previously explored. This gave less number of spontaneous explorations as compared to normal animals. In this study, the chloroform extract and chlorogenic acid significantly decreased the number of alternate arm returns as compared to control. Furthermore, the CHF. Ext and CGA also significantly increased spontaneous alternation performance which was parallel with reported study [47, 48].

Novel object recognition test is use for studying both short and long term memory. The general principle of novel object recognition test is based on exploring new object. The rodents spend more time with unfamiliar object as compared to familiar object. In this study, the CHF. Ext and CGA caused increase exploration of the novel object compared to the familiar object and were comparable to reference drug donepezil. In NORT, the increase of the %DI and novel object exploration time in mice treated with CHF. Ext and CGA suggested improvement of learning and memory. However, Scopolamine significantly decreased the discrimination index, indicating impairment of learning and memory. This effect was reversed by *E. umbellata* CHF. Ext and isolated compound CGA, significantly increased DI and novel object exploration time, indicating that *E. umbellata* fruit possesses memory enhancing activity. These findings were further supported by their potential to inhibit AChE and BChE enzymes both in vitro and in vivo which was also validated by in silico molecular docking study. Current results were in agreement with a previous study of Zahra R et al [48].

The high potency and anti-amnesic potential of CHF. Ext fraction is due to the presence of isolated compound chlorogenic acid which has strong neuroprotective effects on learning and memory impairment that may provide novel leads for the development of AChE inhibitors [27]. CGA is the key polyphenolic constituent that exhibits cardio-protective effects, inhibitory role against lipid hyper oxidation, anti-tumour activity and free radical scavenging potential [49]. Specifically, the anti-anxiety effects of CGA are associated with its anti-oxidant potential that significantly suppresses the second restraint-induced release of serotonin from the rat hippocampus, suggesting that CGA contributes to relaxation of restraint stress [50]. In addition, chlorogenic acid derivatives have shown neuroprotective effects on hydrogen peroxide and amyloid beta (Aβ) - induced cell death in PC12 and SH-SY5Y cells [29].

To the best of our knowledge, there was no reported neuroprotective study on *E. umbellata*. Therefore this is the first study to report important phyto-constituents (chlorogenic acid, ellagic acid, gallic acid and phloroglucinol) in *E. umbellata* fruits which are responsible for both in-vitro and in-vivo anti-cholinesterase activity and in-vivo anti-amnesic potential.

## Conclusion

The *E. umbellata* fruit was subjected to extraction and fractionation. The fractions were tested for different biological potentials. Based on in vitro results chloroform fraction was subjected to isolation of responsible compounds that resulted in isolation of compound **I** to **IV**. Based on literature finding and our in vitro and in vivo results compound**-I** was further investigated to achieve the desire goal. It showed significant antiamnesic activity as assessed by behavioral tests using Y maze and Novel object recognition test in mice animal model. It is concluded that *E. umbellata* is a rich source of potential bioactive compounds which exhibited potent in-vitro and in-vivo neuro-protective activities. *E. umbellata* fruits extract/fractions and isolated compound chlorogenic acid exhibited a cognitive-enhancing effect by reversing scopolamine-induced learning and memory deficits. Thus *E. umbellata* fruits could be recommended for controlling memory impairments and neurological disorders. However, further work is required to investigate their exact mechanism and cellular pathways which are contributing in this cascade.

## Supplementary information

**Additional file 1: Table S1.** Total phenolic and flavonoid contents in extract/fractions of *Elaeagnus umbellata* fruit. **Table S2.** % Choline esterase (AChE and BChE) inhibition potential of extract/fractions of *Elaeagnus umbellata* fruit. **Table S3.** % Cholinesterase (AChE and BChE) inhibition potential of compound I-IV at various concentrations. **Table S4.** % DPPH and ABTS free radical scavenging activity of isolated compounds I-IV. **Table S5.** The chemical shifts of Compound-I in solvent MeOD. **Table S6.** The chemical shifts of compound-IV in solvent MeOD. **Figure S1.** Total Phenolic and Flavonoids Content of CHF.Ext and their linear correlation with anticholinesterase enzymes. **Figure S2.** Structural formulas of isolated compounds I-IV and standard drug donepezil. **Figure S3.** FTIR spectrum of compound-I. **Figure S4.** FAB-Mass of compound-I. **Figure S5.**^1^H- (500 MHz.) NMR spectrum of compound-I. **Figure S6.**^13^C-NMR (126 MHz) spectrum of compound-I. **Figure S7.**^1^H NMR (CHLOROFORM-d, 500MHz) of compound-II. **Figure S8.**^13^C NMR (Chloroform-d, 100MHz) of compound-II. **Figure S9.** EIMS of Compound-II. **Figure S10.**^1^H NMR (METHANOL-d_4_, 500MHz) of compound-III. **Figure S11.**^13^C NMR (METHANOL-d_4_, 100MHz) of compound-III. **Figure S12.** EIMS spectra of compound-III. **Figure S13.** FTIR spectra of compound-IV. **Figure S14.** 1H- (500 MHz) of compound-IV. **Figure S15.**^13^C-NMR (126 MHz) of compound-IV. **Figure S16.** EIMS of compound-IV. **Figure S17.** Escape Latency (seconds) Results of Y-Maze test in different animals groups.

## Data Availability

The data presented in this manuscript belong to the PhD work of Dr. Nausheen Nazir and has not been deposited in any repository yet. However, the materials are available to the researchers upon request.

## Abbreviations

Aq.Ext: Aqueous fraction
AChE: Acetylcholine esterase
ABTS2: 2′-Azino-bis (3-ethylbenzothiazoline-6-sulfonic acid)
AD: Alzheimer’s Disease
Ach: Acetylcholine
But.Ext: *n*-Butanol
BChE: Butyrylcholine esterase
CHF.Ext: Chloroform fraction
C13NMR: Carbon-13 Nuclear Magnetic-resonance
CGA: Chlorogenic acid
DPPH 2: 2-Diphenyl, 1, picrylhydrazyl
*E. umbellata*: *Elaeagnus umbellata*
EtAc.Ext: Ethyl acetate fraction
FTIR: Fourier Transform Infrared-spectroscopy
FAB-MS: Fast Atom Bombardment Mass Spectrum
Hex.Ext: *n*-hexane fraction
HNMR: H^+^-Nuclear Magnetic-resonance
IC_50_: Median inhibitory concentration
i.p.: Intraperitoneal
Met.Ext: hydro methanolic extract
NORT: Novel object recognition Test
p.o.: Per oral
SEM: Standard error mean
STM: Short term memory
SAP: Spontaneous Alternation-performance
