# Supplementary material for: Evaluation of neuroprotective and anti-amnesic effects of Elaeagnus umbellata Thunb. On scopolamine-induced memory impairment in mice
Source: BMC Complement Med Ther. 2020 May 12;20:143. doi: 10.1186/s12906-020-02942-3 (PMC7216467; doi:10.1186/s12906-020-02942-3)
Supplement: Supplementary file 1 — Additional file 1: Table S1. Total phenolic and flavonoid contents in extract/fractions of Elaeagnus umbellata fruit. Table S2. % Choline esterase (AChE and BChE) inhibition potential of extract/fractions of Elaeagnus umbellata fruit. Table S3. % Cholinesterase (AChE and BChE) inhibition potential of compound I-IV at various concentrations. Table S4. % DPPH and ABTS free radical scavenging activity of isolated compounds I-IV. Table S5. The chemical shifts of Compound-I in solvent MeOD. Table S6. The chemical shifts of compound-IV in solvent MeOD. Figure S1. Total Phenolic and Flavonoids Content of CHF.Ext and their linear correlation with anticholinesterase enzymes. Figure S2. Structural formulas of isolated compounds I-IV and standard drug donepezil. Figure S3. FTIR spectrum of compound-I. Figure S4. FAB-Mass of compound-I. Figure S5.1H- (500 MHz.) NMR spectrum of compound-I. Figure S6.13C-NMR (126 MHz) spectrum of compound-I. Figure S7.1H NMR (CHLOROFORM-d, 500MHz) of compound-II. Figure S8.13C NMR (Chloroform-d, 100MHz) of compound-II. Figure S9. EIMS of Compound-II. Figure S10.1H NMR (METHANOL-d4, 500MHz) of compound-III. Figure S11.13C NMR (METHANOL-d4, 100MHz) of compound-III. Figure S12. EIMS spectra of compound-III. Figure S13. FTIR spectra of compound-IV. Figure S14. 1H- (500 MHz) of compound-IV. Figure S15.13C-NMR (126 MHz) of compound-IV. Figure S16. EIMS of compound-IV. Figure S17. Escape Latency (seconds) Results of Y-Maze test in different animals groups. [file 12906_2020_2942_MOESM1_ESM.docx]

**Supplementary material**

**Tables:**

Table S1: Total phenolic and flavonoid contents in extract/fractions of *Elaeagnus umbellata* fruit

| **S.No.** | **Extracts sample** (1000µg/mL) |  | **TPC (** mg GAE/g) | **TFC** (mg QE/g) |
| --- | --- | --- | --- | --- |
| 1 | Met. Ext |  | 26.81 ± 0.33 | 38.94 ± 1.01 |
| 2 | Hex. Ext |  | 36.28 ± 0.69 | 41.05 ± 0.84 |
| 3 | CHF. Ext |  | 56.97 ± 0.77 | 75.53 ± 0.44 |
| 4 | EtAc. Ext |  | 49.15 ± 1.05 | 63.49 ± 1.03 |
| 5 | But. Ext |  | 44.95 ± 0.97 | 52.13 ± 0.68 |
| 6  7 | Aq. Ext  Control |  | 20.54 ± 0.61  1.25 ± 0.55 | 32.06 ± 0.69  1.59 ± 0.25 |

TPC, Total Phenolic Content; TFC, Total Flavonoid Content; Met. Ext, hydro methanolic extract; Hex. Ext, *n*-hexane fraction; CHF. Ext, Chloroform fraction; EtAc. Ext, Ethyl acetate fraction; But. Ext, *n-*Butanol; Aq. Ext, Aqueous fraction. Note: Total phenolic content expressed as gallic acid equivalents (mg GAE)/g plant extract; B) Total flavonoid content expressed as quercetin equivalents (mg QE)/g plant extract**.** Data are presented as mean ± SEM, n=3 experiments, Values are significantly different as compare to control, *P < 0.05, **P < 0.01, ***P < 0.001.

Table S2: % Choline esterase (AChE and BChE) inhibition potential of extract/fractions of *Elaeagnus umbellata* fruit

| S.No | Extract/fractions | Concentration  (µg/mL) | % AChE | AChE  IC_50_ µg/mL | % BChE | BChE  IC_50_ µg/mL |
| --- | --- | --- | --- | --- | --- | --- |
|  |  |  | **Mean ± SEM** |  | **Mean ± SEM** |  |
| 1 | Met. Ext | 1000 | 77± 0.5*** | 64 | 83±0.5*** | 55 |
|  |  | 500 | 70± 1.0*** |  | 75±0.4*** |  |
|  |  | 250 | 66± 1.1*** |  | 62±0.6*** |  |
|  |  | 125 | 57± 0.5*** |  | 53±1.3*** |  |
|  |  | 62.5 | 49± 0.3*** |  | 48±0.5*** |  |
|  |  | 31.05 | 44± 1.2*** |  | 43±1.2*** |  |
| 2 | Hex. Ext | 1000 | 70±0.2*** | 69 | 69±0.6*** | 65 |
|  |  | 500 | 65±1.0*** |  | 62±0.3*** |  |
|  |  | 250 | 58±0.5*** |  | 57±0.5*** |  |
|  |  | 125 | 52±1.0*** |  | 50±1.0*** |  |
|  |  | 62.5 | 48±0.5*** |  | 44±0.5*** |  |
|  |  | 31.05 | 41±0.4*** |  | 35±1.0*** |  |
| 3 | CHF. Ext | 1000 | 87±1.2*** | 33 | 86±0.3* | 35 |
|  |  | 500 | 78±0.5*** |  | 79±1.0*** |  |
|  |  | 250 | 74±0.4*** |  | 71±0.5*** |  |
|  |  | 125 | 67±0.5*** |  | 60±1.1*** |  |
|  |  | 62.5 | 61±0.4 ^ns^ |  | 55±0.5*** |  |
|  |  | 31.05 | 49±0.5*** |  | 48±0.2*** |  |
| 4 | EtAc. Ext | 1000 | 84±1.0 *** | 55 | 82±0.5*** | 58 |
|  |  | 500 | 72±1.1*** |  | 76±1.1*** |  |
|  |  | 250 | 69±0.5*** |  | 68±0.5*** |  |
|  |  | 125 | 60±0.4*** |  | 64±0.6*** |  |
|  |  | 62.5 | 50±0.3*** |  | 50±0.4*** |  |
|  |  | 31.05 | 42±0.4*** |  | 46±0.4*** |  |
| 5 | But. Ext | 1000 | 67±0.5*** | 90 | 70±0.5*** | 62 |
|  |  | 500 | 60±0.3*** |  | 65±0.5*** |  |
|  |  | 250 | 54±0.4*** |  | 59±0.4*** |  |
|  |  | 125 | 49±1.1*** |  | 54±1.1*** |  |
|  |  | 62.5 | 43±0.3*** |  | 49±0.4*** |  |
|  |  | 31.05 | 34±1.1*** |  | 40±0.3*** |  |
| 6 | Aq. Ext | 1000 | 70±1.1*** | 74 | 71±1.0*** | 78 |
|  |  | 500 | 65±1.0*** |  | 68±1.0*** |  |
|  |  | 250 | 59±0.2*** |  | 61±1.1*** |  |
|  |  | 125 | 54±0.5*** |  | 51±0.5*** |  |
|  |  | 62.5 | 46±0.4*** |  | 40±0.5*** |  |
|  |  | 31.05 | 39±0.4*** |  | 38±0.4*** |  |
| 7 | Standard  Donepezil | 1000 | 93±0.5 | 25 | 92±0.3 | 28 |
|  |  | 500 | 89±1.1 |  | 90±0.2 |  |
|  |  | 250 | 81±0.5 |  | 86±0.5 |  |
|  |  | 125 | 78±0.4 |  | 78±1.1 |  |
|  |  | 62.5 | 66±0.3 |  | 69±0.5 |  |
|  |  | 31.05 | 54±0.5 |  | 53±0.4 |  |

AChE, Acetyl cholinesterase; BChE, Butyrylcholinesterase; Met. Ext, hydro methanolic extract; Hex. Ext, *n*-hexane fraction; CHF. Ext, Chloroform fraction; EtAc. Ext, Ethyl acetate fraction; But. Ext, *n-*Butanol; Aq. Ext, Aqueous fraction

Table S3: % Cholinesterase (AChE and BChE) inhibition potential of compound I-IV at various concentrations

| S.No | Sample | Concentration  (µg/mL) | % AChE | AChE  IC_50_ µg/mL | % BChE | BChE  IC_50_ µg/mL |
| --- | --- | --- | --- | --- | --- | --- |
|  |  |  | **Mean ± SEM** |  | **Mean ± SEM** |  |
| 1 | Compound **-I** | 1000 | 90±0.2^ns^ | 31 | 88±0.2 ^ns^ | 32 |
|  |  | 500 | 82±0.1*** |  | 85±0.4** |  |
|  |  | 250 | 76±0.5* |  | 76±0.2*** |  |
|  |  | 125 | 68±0.4*** |  | 70±0.5*** |  |
|  |  | 62.5 | 59±0.3*** |  | 62±0.6*** |  |
|  |  | 31.05 | 50±0.4 ^ns^ |  | 50±0.4 ^ns^ |  |
| 2 | Compound –**II** | 1000 | 81±0.5** | 59 | 80±0.5*** | 62 |
|  |  | 500 | 75±0.4*** |  | 74±0.2*** |  |
|  |  | 250 | 68±0.1*** |  | 68±0.4*** |  |
|  |  | 125 | 52±0.4*** |  | 60±0.5*** |  |
|  |  | 62.5 | 48±0.5*** |  | 52±0.3*** |  |
|  |  | 31.05 | 41±0.3** |  | 45±0.4*** |  |
| 4 | Compound –**III** | 1000 | 76±2.1*** | 45 | 66±1.5*** | 83 |
|  |  | 500 | 62±1.2*** |  | 61±2.5*** |  |
|  |  | 250 | 55±0.3*** |  | 54±1.1*** |  |
|  |  | 125 | 49±1.4*** |  | 47±0.5*** |  |
|  |  | 62.5 | 43±1.1*** |  | 42±1.1*** |  |
|  |  | 31.05 | 39±0.5*** |  | 36±0.5*** |  |
| 6 | Compound -**IV** | 1000 | 66±1.2*** | 92 | 58±2.2*** | 115 |
|  |  | 500 | 52±1.6*** |  | 50±2.1*** |  |
|  |  | 250 | 40±0.5*** |  | 47±0.5*** |  |
|  |  | 125 | 34±1.5*** |  | 41±1.1*** |  |
|  |  | 62.5 | 29±2.1*** |  | 35±2.1*** |  |
|  |  | 31.05 | 21±1.5*** |  | 29±0.5*** |  |
| 9 | Standard  Donepezil | 1000 | 93±0.5 | 25 | 92±0.3 | 26 |
|  |  | 500 | 89±1.1 |  | 90±0.2 |  |
|  |  | 250 | 81±0.5 |  | 86±0.5 |  |
|  |  | 125 | 78±0.4 |  | 78±1.1 |  |
|  |  | 62.5 | 66±0.3 |  | 69±0.5 |  |
|  |  | 31.05 | 63±0.5 |  | 65±0.4 |  |

AChE, Acetyl cholinesterase; BChE, Butyrylcholinesterase

Table S4: % DPPH and ABTS free radical scavenging activity of isolated compounds I-IV

| S.No | Sample | | Concentration  (µg/mL) | | \| % DPPH Scavenging \| \| --- \|   Mean ± SEM | | DPPH  IC_50_ µg/mL | | \| % ABTS Scavenging \| \| --- \|   Mean ± SEM | | ABTS  IC_50_ µg/mL | |  |
| --- | --- | --- | --- | --- | --- | --- | --- | --- | --- | --- | --- | --- | --- | --- | --- |
| 1 | | Compound-**I** | | 1000 | | 80±0.4 *** | | 35 | | 78±0.5 *** | | 38 | |
|  |  |  |  | 500 | | 70±1.1 *** | |  |  | 75±0.3*** | |  |  |
|  |  |  |  | 250 | | 66±0.4 *** | |  |  | 69±1.1 *** | |  |  |
|  |  |  |  | 125 | | 60±0.3*** | |  |  | 65±0.6 ** | |  |  |
|  |  |  |  | 62.5 | | 53±0.5*** | |  |  | 60±1.0 * | |  |  |
|  |  |  |  | 31.05 | | 53±1.1 *** | |  |  | 55±0.5 ^ns^ | |  |  |
| 2 | | Compound-**II** | | 1000 | | 77±1.1*** | | 39 | | 69±1.0*** | | 44 | |
|  |  |  |  | 500 | | 70±0.5*** | |  |  | 65±0.4*** | |  |  |
|  |  |  |  | 250 | | 60±1.2** | |  |  | 64±0.3*** | |  |  |
|  |  |  |  | 125 | | 59±0.4*** | |  |  | 57±1.0*** | |  |  |
|  |  |  |  | 62.5 | | 57±0.5*** | |  |  | 49±1.0 *** | |  |  |
|  |  |  |  | 31.05 | | 44±1.4*** | |  |  | 41±0.5 *** | |  |  |
| 4 | | Compound-**III** | | 1000 | | 76±0.5*** | | 52 | | 65±2.1 *** | | 54 | |
|  |  |  |  | 500 | | 70±1.1*** | |  |  | 60±0.5 *** | |  |  |
|  |  |  |  | 250 | | 59±1.5*** | |  |  | 54±1.1 *** | |  |  |
|  |  |  |  | 125 | | 50±1.2*** | |  |  | 50±1.5 *** | |  |  |
|  |  |  |  | 62.5 | | 48±0.5*** | |  |  | 48±0.4 *** | |  |  |
|  |  |  |  | 31.05 | | 43±1.4*** | |  |  | 43±1.3 *** | |  |  |
| 6 | | Compound -**IV** | | 1000 | | 67±0.4*** | | 105 | | 54±1.1 *** | | 112 | |
|  |  |  |  | 500 | | 57±1.0*** | |  |  | 51±0.5 *** | |  |  |
|  |  |  |  | 250 | | 51±1.0*** | |  |  | 45±1.0 *** | |  |  |
|  |  |  |  | 125 | | 40±0.5*** | |  |  | 39±1.2 *** | |  |  |
|  |  |  |  | 62.5 | | 36±1.1*** | |  |  | 33±0.5 *** | |  |  |
|  |  |  |  | 31.05 | | 32±0.5*** | |  |  | 25±1.0 *** | |  |  |
| 9 | | Ascorbic acid | | 1000 | | 91±0.5 | | 29 | | 90±0.5 | | 32 | |
|  |  |  |  | 500 | | 86±1.0 | |  |  | 84±1.1 | |  |  |
|  |  |  |  | 250 | | 80±0.4 | |  |  | 78±0.5 | |  |  |
|  |  |  |  | 125 | | 77±0.4 | |  |  | 70±0.5 | |  |  |
|  |  |  |  | 62.5 | | 73±0.5 | |  |  | 64±1.0 | |  |  |
|  |  |  |  | 31.05 | | 69±0.4 | |  |  | 56±1.1 | |  |  |

Table S5: The chemical shifts of Compound-I in solvent MeOD

| Carbon No. | ^1^H- (δ) | ^13^C- (δ) | Multiplicity |
| --- | --- | --- | --- |
| 1 | - | 76.11 | C |
| 2α | 2.04 | 38.13 | CH_2_ |
| 2β | 2.18 |  |  |
| 3 | 4.18 | 71.27 | CH |
| 4 | 3.74 | 73.45 | CH |
| 5 | 5.34 | 71.89 | CH |
| 6α | 2.24 | 38.75 | CH_2_ |
| 6β | 2.07 |  |  |
| 7 | - | 176.99 | C |
| 1´ | - | 127.78 | C |
| 2´ | 7.05 | 115.19 | CH |
| 3´ | - | 146.68 | C |
| 4´ | - | 149.46 | C |
| 5´ | 6.78 | 116.49 | CH |
| 6´ | 6.95 | 122.97 | CH |
| 7´ | 7.56 | 147.04 | CH |
| 8´ | 6.26 | 115.17 | CH |
| 9´ | ---- | 168.70 | C |
| Solvent | 3.74 | 48.98 | - |

Table S6: The chemical shifts of compound-IV in solvent MeOD

| Carbon No. | ^1^H (δ) | ^13^C (δ) | Multiplicity |
| --- | --- | --- | --- |
| 1 | - | 160.0 | C H |
| 2 | 5.83 | 95.5 | CH_2_ |
| 3 | - | 160.0 | C H |
| 4 | 5.83 | 95.5 | CH_2_ |
| 5 | - | 160.0 | CH |
| 6 | 5.83 | 95.5 | CH_2_ |
| OH | - | 9.11 | - |
| Solvent | 3.74 | 48.98 | - |

**Figures:**

**
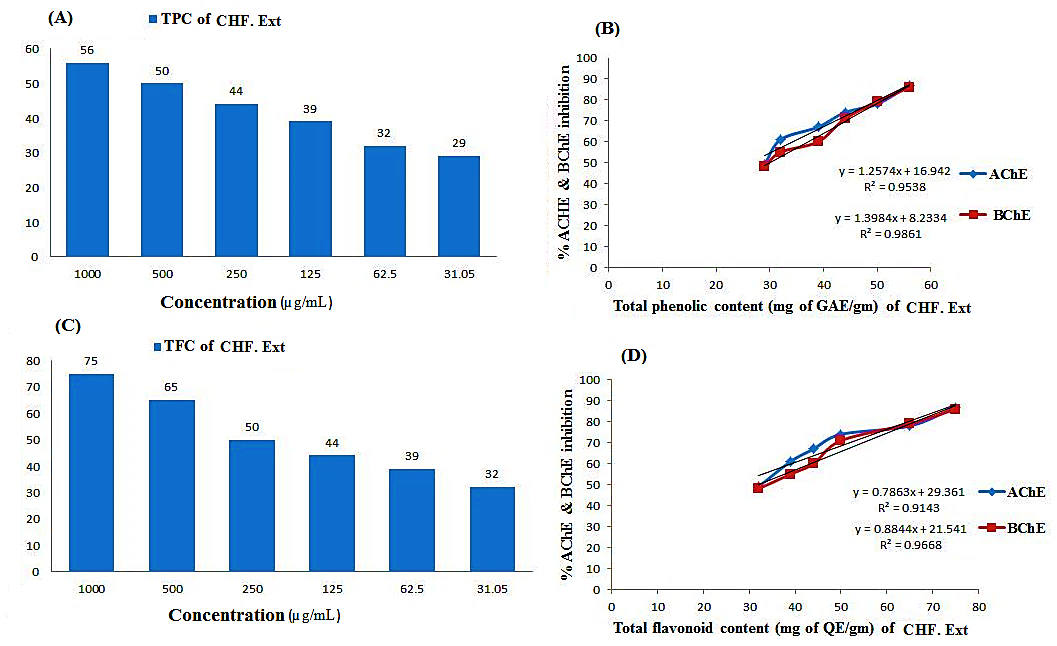
**

**Figure S1:** Total Phenolic and Flavonoids Content of CHF.Ext and their linear correlation with anticholinesterase enzymes **{(A)** Total Phenolic contents (TPC) of CHF.Ext at various concentration **(B)** Linear correlation of TPC of CHF.Ext vs. % AChE and %BChE inhibition **(C)** Total Flavonoids Content (TFC) of CHF.Ext at various concentration **(D)** TFC vs. Percent acetyl cholinesterase **(**AChE) and buterly cholinesterase (BChE) inhibition}


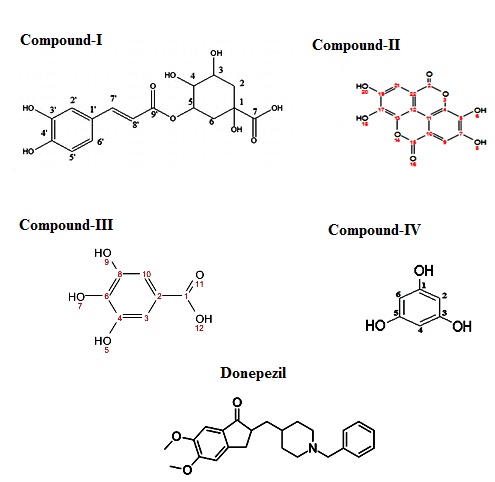


**Figure S2:** Structural formulas of isolated compounds **I-IV** and standard drug donepezil

**
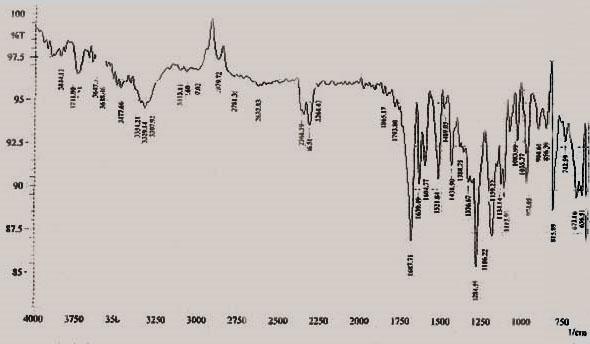
**

**Figure S3:** FTIR spectrum of compound-**I**

**
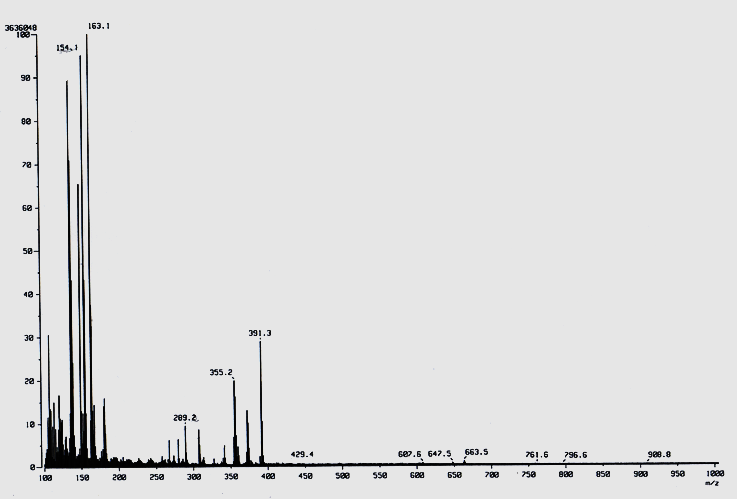
**

**Figure S4:** FAB-Mass of compound-**I**

**
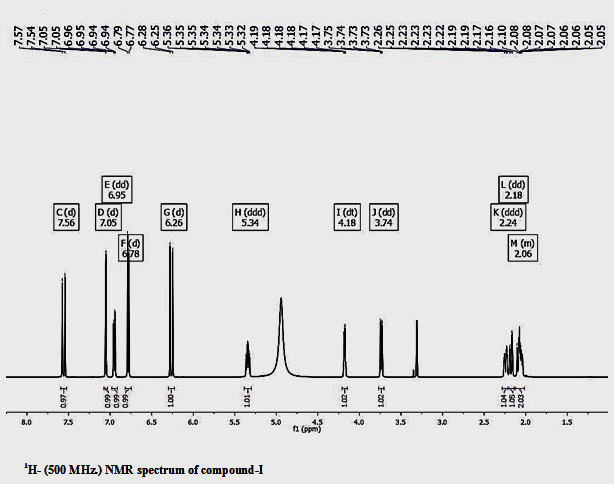
**

**Figure S5:**^1^H- (500 MHz.) NMR spectrum of compound-**I**

**
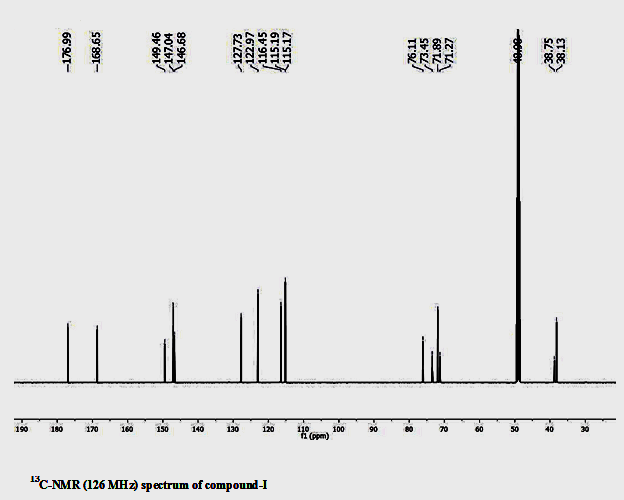
**

**Figure S6:**^13^C-NMR (126 MHz) spectrum of compound-**I**

**
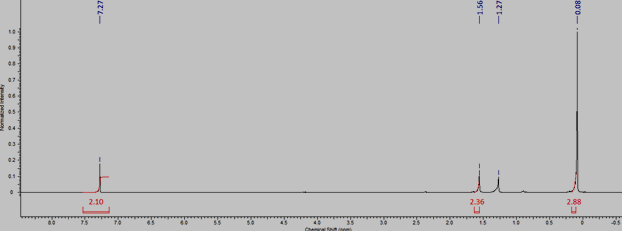
**

**Figure S7:**^1^H NMR (CHLOROFORM-d, 500MHz) of compound-**II**

**
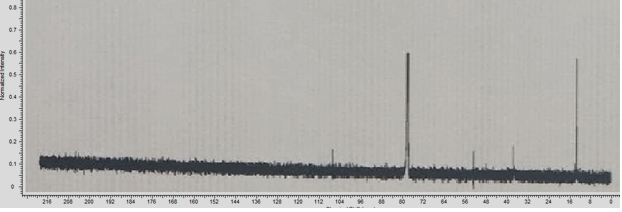
**

**Figure S8:**^13^C NMR (Chloroform-d, 100MHz) of compound-**II**

**
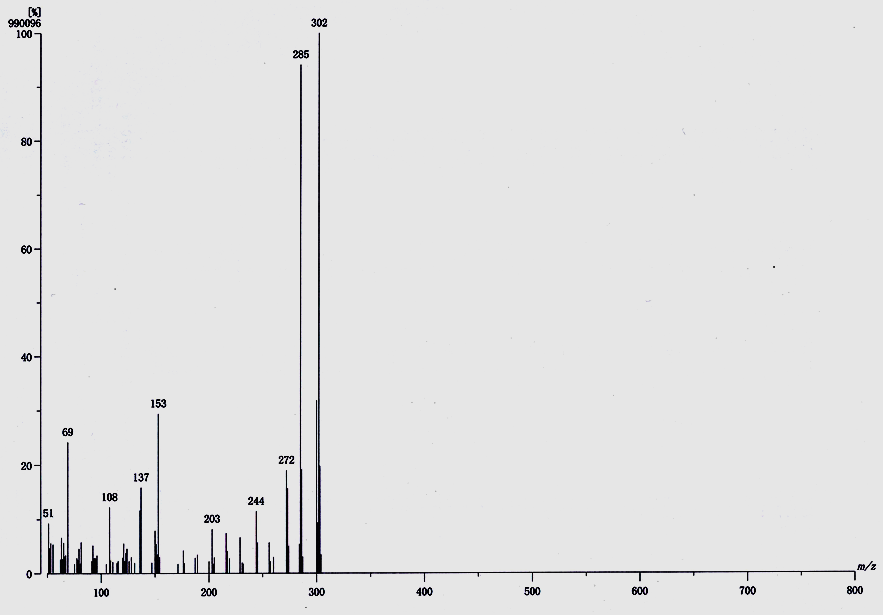
**

**Figure S9:** EIMS of Compound-**II**

**
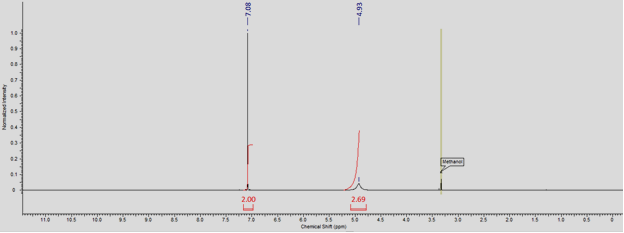
**

**Figure S10:**^1^H NMR (METHANOL-d_4_, 500MHz) of compound-**III**

**
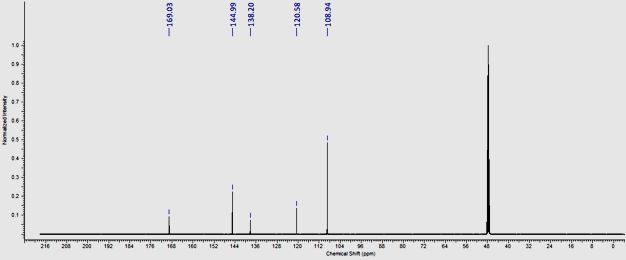
**

**Figure S11:**^13^C NMR (METHANOL-d_4_, 100MHz) of compound-**III**

**
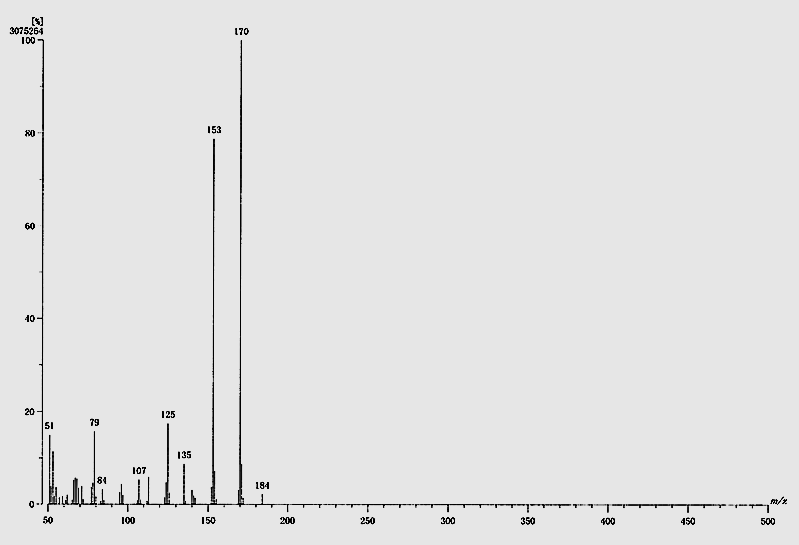
**

**Figure S12:** EIMS spectra of compound-**III**

**
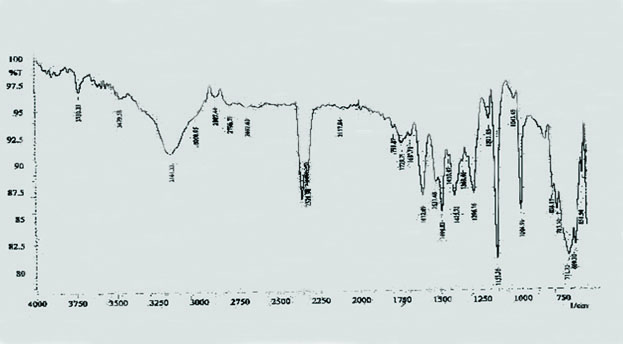
**

**Figure S13:** FTIR spectra of compound-**IV**

**
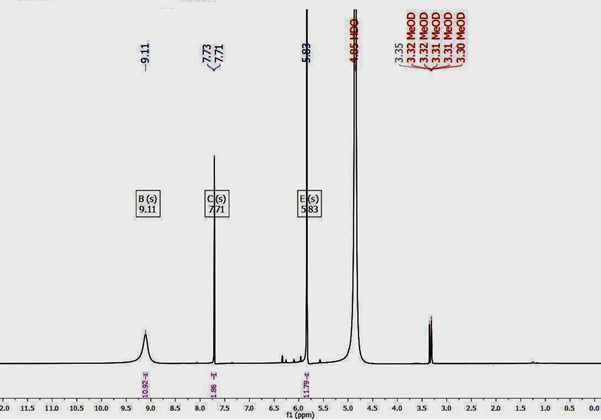
**

**Figure S14:** 1H- (500 MHz) of compound-**IV**

**
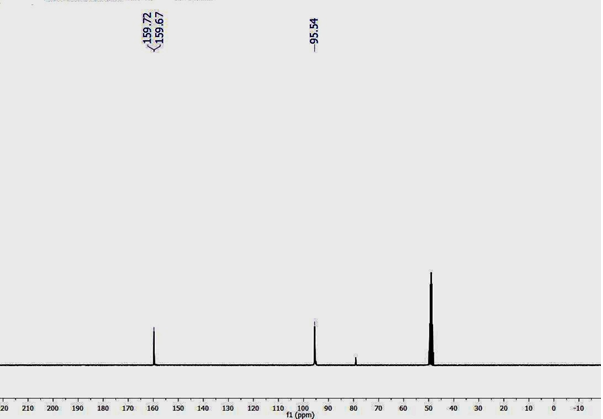
**

**Figure S15:**^13^C-NMR (126 MHz) of compound-**IV**

**
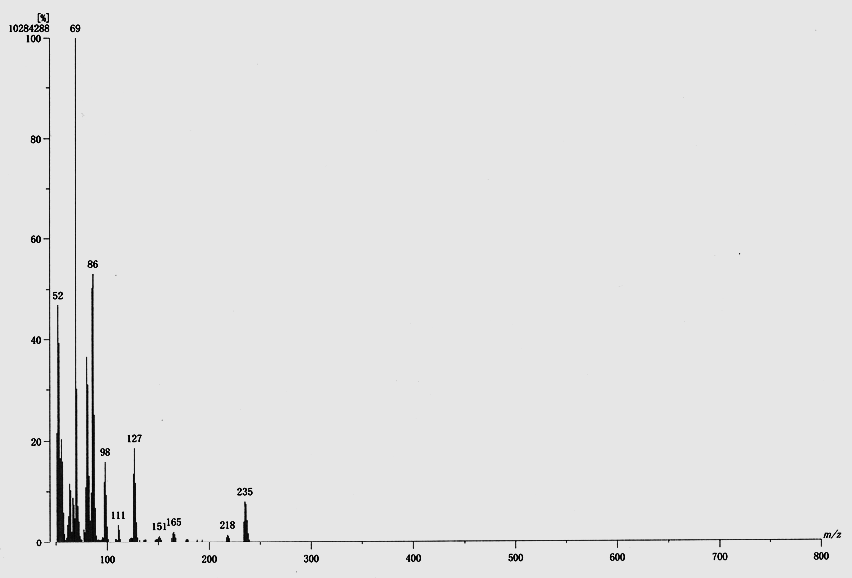
**

**Figure S16:** EIMS of compound-**IV**

**
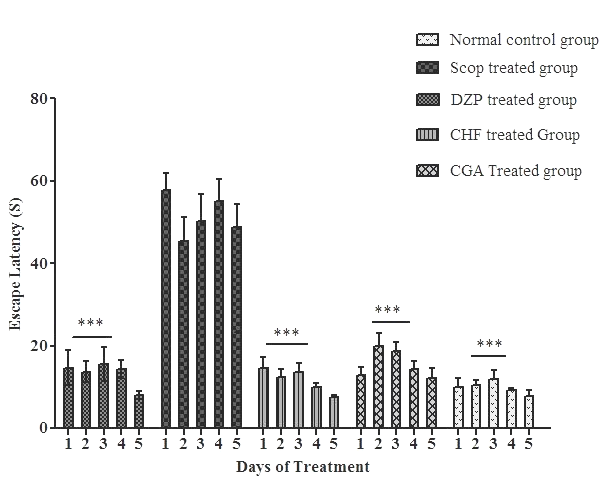
**

**Figure S17:** Escape Latency (seconds) Results of Y-Maze test in different animals groups. (*** indicates that values were significantly different, p< 0.001)
